# Identification and characterization of multiple rubisco activases in chemoautotrophic bacteria

**DOI:** 10.1038/ncomms9883

**Published:** 2015-11-16

**Authors:** Yi-Chin Candace Tsai, Maria Claribel Lapina, Shashi Bhushan, Oliver Mueller-Cajar

**Affiliations:** 1 School of Biological Sciences, Nanyang Technological University, 60 Nanyang Drive, Singapore 637551. Singapore

## Abstract

Ribulose-1,5-bisphosphate carboxylase/oxygenase (rubisco) is responsible for almost all biological CO_2_ assimilation, but forms inhibited complexes with its substrate ribulose-1,5-bisphosphate (RuBP) and other sugar phosphates. The distantly related AAA+ proteins rubisco activase and CbbX remodel inhibited rubisco complexes to effect inhibitor release in plants and α-proteobacteria, respectively. Here we characterize a third class of rubisco activase in the chemolithoautotroph *Acidithiobacillus ferrooxidans*. Two sets of isoforms of CbbQ and CbbO form hetero-oligomers that function as specific activases for two structurally diverse rubisco forms. Mutational analysis supports a model wherein the AAA+ protein CbbQ functions as motor and CbbO is a substrate adaptor that binds rubisco via a von Willebrand factor A domain. Understanding the mechanisms employed by nature to overcome rubisco's shortcomings will increase our toolbox for engineering photosynthetic carbon dioxide fixation.

## Abstract (web summary)

The CO_2_-fixing enzyme rubisco requires motor proteins known as rubisco activases to remove inhibitors bound to its active site. Here the authors describe a new class of rubisco activase present in chemoautotrophic bacteria that belongs to the MoxR family of AAA+ ATPases.

## Graphical abstract

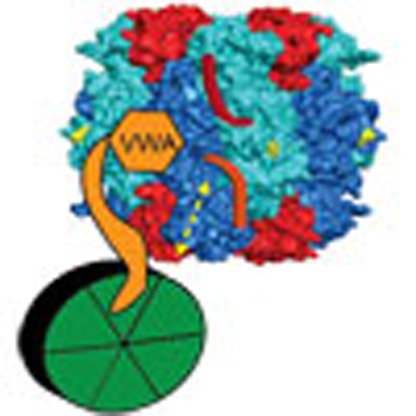

The key photosynthetic CO_2_-fixing enzyme ribulose-1,5-bisphosphate carboxylase/oxygenase (rubisco) is renowned for its slow kinetics and poor substrate specificity, making it a long-standing target for crop improvement efforts^1^,^2^. It is accepted that by taking advantage of the natural variability existing in rubisco kinetic properties the efficiency of photosynthesis could be improved^3^. However, so far attempts to produce ‘superior' enzymes in plant chloroplasts have been foiled by an incomplete appreciation of the complex suite of accessory proteins that govern rubisco's biogenesis and maintenance^4^,^5^. Hence a thorough understanding of such factors will be essential^6^,^7^,^8^.

Plants encode one highly conserved so-called green-type form I enzyme consisting of eight ∼52 kDa large subunits and eight ∼15 kDa small subunits (L_8_S_8_)^9^. In contrast, proteobacteria often possess a multitude of diverse rubiscos, including both red- and green-type form I enzymes (L_8_S_8_) and form II rubisco, which lacks small subunits and generally forms an (L_2_)_*X*_ oligomer^10^,^11^.

All rubiscos have a propensity to catalyse a side reaction with oxygen, which necessitates the energetically wasteful photorespiratory pathway^12^. One strategy that evolved to minimize the oxygenation reaction involved increasing the CO_2_/O_2_ specificity, albeit at the cost of a reduced catalytic turnover rate^13^. The increased structural rigidity associated with higher specificity rubiscos correlates with a tendency of the enzyme to form dead-end inhibited complexes with sugar phosphates^14^,^15^. Physiologically relevant inhibitors include the substrate ribulose-1,5-bisphosphate (RuBP), misfire products such as xylulose-1,5-bisphosphate, or regulatory compounds such as carboxyarabinitol 1-phosphate^16^.

To become functional, a conserved active-site lysine needs to become carbamylated by a non-substrate CO_2_, followed by co-ordination of a Mg^2+^ ion^17^. Inhibitors bind to the active sites of both the activated (ECM) and inactive form (E) of the enzyme. To maintain photosynthetic CO_2_ fixation a group of molecular motors known as the rubisco activases evolved to conformationally remodel inhibited rubisco complexes leading to release of the inhibitors. So far two distantly related AAA+ (ATPases associated with various cellular activities) proteins, rubisco activase (Rca) and CbbX have been described to act on plant green-type and α-proteobacterial red-type form I rubisco respectively^7^,^18^,^19^,^20^,^21^. Like most AAA+ proteins both Rca and CbbX can form ring-shaped hexamers, but function via distinct mechanisms. The current model for CbbX function involves destabilization of the inhibited rubisco active site via transient threading of the rubisco large subunit C terminus through the pore of the hexamer, analogous to the mechanism of the intensely studied AAA+ proteins ClpB or ClpX^22^,^23^. Whereas CbbX function is completely dependent on a conserved pore-loop tyrosine and the rubisco large subunit C terminus, Rca does not possess a typical pore-loop 1 and activase activity is tolerant of rubisco large subunit C-terminal modifications^24^. Instead an interaction between two surface-displayed large subunit residues (position 89 and 94) and a helix insertion in the α-helical subdomain of the activase has been established^20^,^25^,^26^,^27^. Nevertheless, mutational analysis of predicted pore loops does support a role in Rca function^20^. Following active-site remodelling, the released inhibitors can then be degraded by specific sugar phosphatases^28^,^29^.

Chemoautotrophic proteobacteria that oxidize sulphur and other inorganic substrates use rubisco to perform ‘dark' CO_2_ fixation in diverse habitats including the dark ocean^30^, but their genomes generally do not encode Rca or CbbX. Instead two open-reading frames, *cbbQ* and *cbbO* are regularly found directly downstream of the rubisco genes in multiple operons^11^ (Fig. 1a). Co-expressing rubisco with CbbQ and/or CbbO in *Escherichia coli* was reported to lead higher rubisco activities in cell extracts and more active purified rubisco, suggesting the proteins' involvement in some form of posttranslational rubisco modification^31^,^32^. Subsequent similar co-expression experiments did not observe such effects^33^,^34^,^35^.

*CbbQ* sequences are highly conserved (Supplementary Fig. 1) and belong to the large, but poorly studied MoxR group of AAA+ ATPases. CbbQ proteins are very short (∼260 residues), essentially comprising a single AAA+ module. The MoxR group is widely distributed in Archaea and bacteria and believed to represent a new class of molecular chaperones^36^,^37^,^38^. Incidentally, the best-studied MoxR protein, RavA, has been shown to relieve inhibition of lysine decarboxylase by the small-molecule ppGpp^37^. In many cases this group of proteins is associated with a downstream gene encoding a protein containing a von Willebrand Factor A (VWA) domain. Accordingly the ∼770 residue CbbO proteins contain a C-terminal VWA domain with a perfect metal-ion-dependent adhesion site (MIDAS)^39^ (Supplementary Fig. 2). This well-studied domain generally mediates protein–protein interactions via a carboxylate side-chain^40^,^41^. The sequence preceding the VWA domain (∼550 residues) is poorly conserved and has no discernable homology to any characterized proteins^33^.

Here we demonstrate using pure components that CbbQ and CbbO isoforms form hetero-oligomeric complexes that function as genuine and specific activases of two distinct rubisco enzymes. Extensive site-directed mutagenesis indicates a conserved mechanism of activation exhibiting both similarities and differences to the Rca and CbbX systems. Understanding the diversity of accessory proteins that has evolved to overcome intrinsic limitations of the key enzyme rubisco will empower synthetic biology approaches aimed at increasing the productivity of crops and other photosynthetic systems.

## Results

### CbbQ and CbbO isoforms form hetero-oligomeric complexes

**Figure 1:**
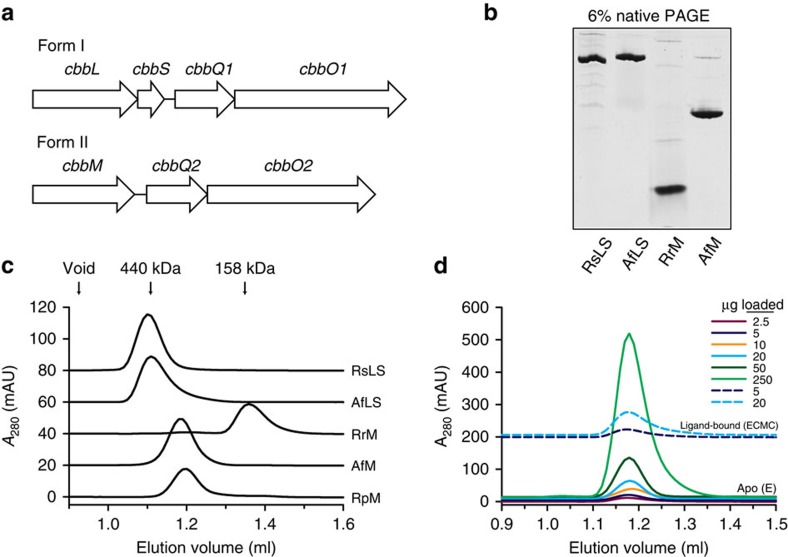
The oligomeric state of *A. ferrooxidans* rubisco proteins. (**a**) Arrangement of the CO_2_-fixation operons encoding form I and form II rubisco in *A. ferrooxidans*. (**b**) Native PAGE analysis of purified AfLS and AfM complexes compared to hexadecameric *Rhodobacter sphaeroides* form I (RsLS) and dimeric *Rhodospirillum rubrum* form II (RrM) rubisco. (**c**) Determination of native molecular weight of rubisco enzymes by analytical gel filtration. RpM, *Rhodopseudomonas palustris* form II rubisco (hexameric)^10^. Ten microgram of protein was loaded per experiment. (**d**) Gel-filtration analysis of AfM at varying concentrations in the apo form (E) and in the CABP bound form (ECMC).

We wanted to test the hypothesis that the *cbbQ* and *cbbO* genes encode a third convergently evolved rubisco activation system. Therefore, *E. coli* was used to recombinantly produce the proteins encoded by form I and form II rubisco operons in *Acidithiobacillus ferrooxidans* (Fig. 1a).

Pure form I (AfLS) and form II rubisco (AfM) was obtained in functional form. Gel filtration and native PAGE supported a typical L_8_S_8_ hexadecamer for AfLS, whereas AfM appeared significantly larger than the dimeric form II rubisco from *Rhodospirillum rubrum* (RrM) (Fig. 1b,c). Satagopan *et al.*^10^ have recently described a form II rubisco from *Rhodopseudomonas palustris* (RpM) that crystallized as a hexamer. We found that RpM and AfM behaved identically using gel-filtration and sedimentation analysis (Fig. 1c, Supplementary Figs 3 and 4). Using a combined Stokes radius/sedimentation analysis^42^,^43^ a mass of 313 kDa (AfM) and 308 kDa (RpM) was calculated, supporting a hexameric oligomeric state for both enzymes (Table 1). The elution volume of AfM from the sizing column was independent of protein concentration or whether the tight-binding inhibitor carboxyarabinitol-1,5-bisphosphate (CABP)^44^ was bound (Fig. 1d). These results indicate that in solution AfM occupies a single well-defined oligomeric state, in contrast to the homologue from *Methanococcoides burtonii*, which has been shown to populate multiple oligomeric states^45^.

**Figure 2:**
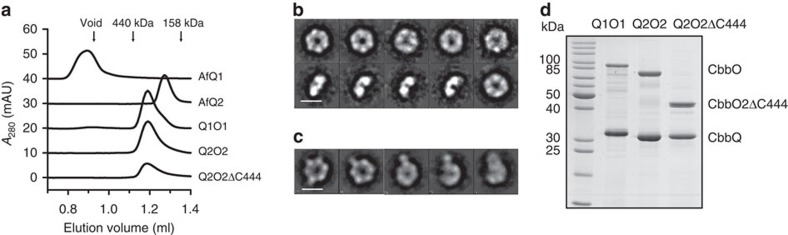
Analysis of the CbbQO hetero-oligomers. (**a**) Gel-filtration analysis of the recombinant, purified CbbQ and CbbQO complexes. Ten microgram of protein was loaded per experiment. (**b**,**c**) Characteristic (**b**) and selected (**c**) unbiased 2D class averages of AfQ2 (**b**) and Q2O2ΔC444 (**c**) incubated with 5 mM Mg-ATP. Scale bar, 100 Å. (**d**) SDS–PAGE analysis of Q1O1, Q2O2 and Q2O2ΔC444 (4 μg protein loaded per lane).

Whereas it was possible to purify both isoforms of CbbQ separately (AfQ1 and AfQ2), CbbO isoforms were insoluble unless co-expressed with their respective CbbQ isoform (Supplementary Fig. 5). Both CbbQ and CbbO isoforms were produced as His_6_–Ubiquitin fusion proteins^46^, which resulted in higher yields of soluble protein. This approach permitted an initial immobilized metal ion affinity chromatography capture step to be incorporated. Precise cleavage of the ubiquitin moiety was possible using a specific protease^46^. Following cleavage, a significant proportion of the recombinant protein precipitated in all cases, however, the soluble fraction could be purified further using ion-exchange chromatography and gel filtration (A representative purification of Q2O2 is shown in Supplementary Fig. 6a). CbbQ and CbbO co-purified using ion-exchange and size-exclusion chromatography indicating the formation of a stable hetero-oligomeric complex (Fig. 2a). To test whether the presence of nucleotide altered the relative proportion of CbbQ and CbbO subunits in the isolated complexes, we performed a small-scale purification of Q2O2, which included 1 mM Mg-ATP in all buffers. The purification profile was identical in the presence and absence of nucleotide (Supplementary Fig. 6b).

In EM, AfQ2 appeared as ring-shaped particles displaying a hexameric arrangement typical for AAA+ ATPases (Fig. 2b, Supplementary Fig. 7), and Stokes radius/sedimentation analysis yielded a molecular mass of 173 kDa, consistent with a monodisperse hexamer (Fig. 2a, Supplementary Figs 3 and 4, Table 1). AfQ1 eluted in the void volume of the sizing column and likely formed soluble aggregates (Fig. 2a). EM of Q1O1 and Q2O2 resulted in heterogeneous particles that could not be processed (data not shown). However, EM analysis of a complex formed with AfQ2 and C-terminally truncated O2 (Q2O2ΔC444, residues 1–444) allowed identification and selection of particles (Supplementary Fig. 8a,b). Reference-free 2D classification revealed an AfQ2 ring-shaped particle with an additional density emerging radially from the ring in a fraction of unbiased class averages (Fig. 2c), consistent with the interaction of a AfQ2 hexamer with the O2ΔC444 fragment. Both combined Stokes radius/sedimentation analysis (Fig. 2a, Supplementary Figs 3 and 4, Table 1) and quantitative densitometry (Fig. 2d and Supplementary Fig. 8c) supported a CbbQ_6_CbbO_1_ stoichiometry for Q1O1 and Q2O2. Densitometry of Q2O2ΔC444 was not consistent with this stoichiometry, but yielded a ratio of 6 CbbQ subunits to 3.5 CbbO fragments assuming equal binding of the Coomassie dye (Supplementary Fig. 8c). In contrast the hydrodynamic analysis of Q2O2ΔC444 supported the expected stoichiometry, yielding a molecular weight of 218 kDa (predicted: 231 kDa; Table 1, Fig. 2a, Supplementary Figs 3 and 4). In summary our results indicate that one CbbO subunit interacts with the ring-shaped hexamer via its poorly conserved N-terminal region (residues 1–444).

### The CbbQO complexes function as rubisco activases

**Figure 3:**
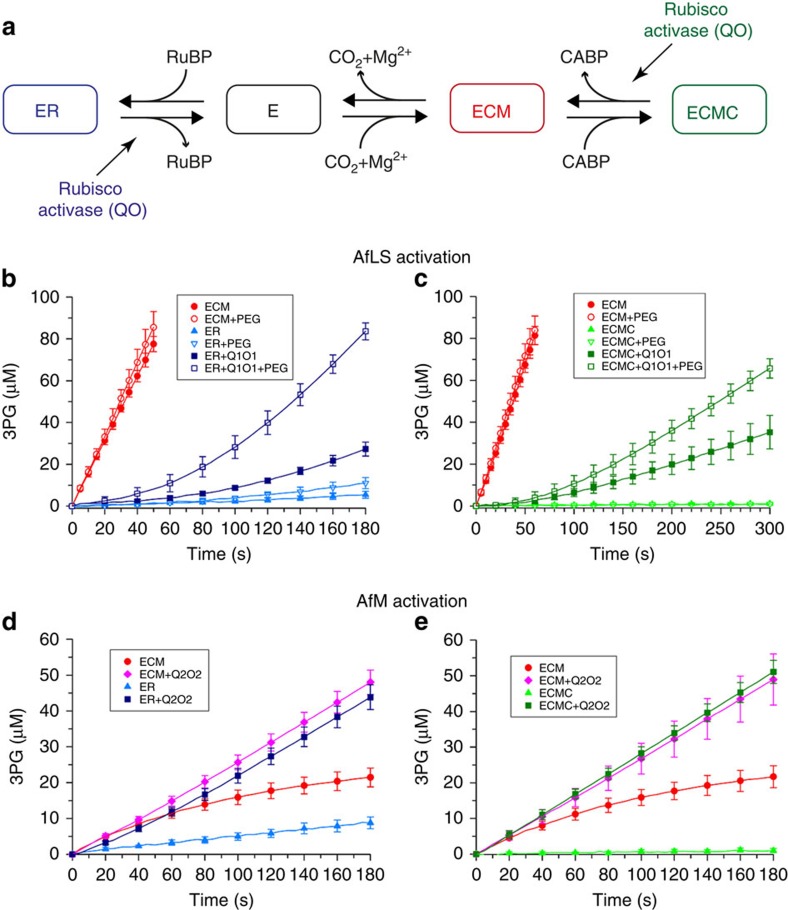
The CbbQO complexes function as rubisco activases. (**a**) The relationship between rubisco complexes used in this study. The apoenzyme E needs to bind CO_2_ and Mg^2+^ cofactors to form the active ECM. Both E and ECM can be inactivated by binding to RuBP (forming ER) or CABP (forming ECMC), respectively. Rubisco activases release inhibitors favouring ECM formation. (**b**–**e**) The CbbQO complexes activate their respective rubisco enzymes. Rubisco activity assays of activated (ECM) and inactivated (ER and ECMC) AfLS (0.3 μM active sites, 20 mM NaHCO_3_) (**b**,**c**) or AfM (0.1 μM active sites, 5 mM NaHCO_3_) complexes (**d**,**e**) in the absence and presence of Q1O1 (**b**,**c**) or Q2O2 (**d**,**e**) (0. 27 μM oligomer). PEG indicates addition of 5% v/v polyethylene glycol 3350. Error bars indicate the mean and s.d. of at least three independent experiments.

For each rubisco form we prepared and compared the activities of three forms: the fully activated enzyme (ECM); the apoenzyme inhibited by its substrate RuBP (ER); and the activated enzyme bound to the tight-binding transition state analogue CABP (ECMC)^44^ (Fig. 3a). As expected for an activase-requiring rubisco, the form I (AfLS) ER and ECMC complexes were inactive and ECM displayed a linear activity corresponding to 5.2 s^−1^ (Fig. 3b,c). When either ER or ECMC was assayed in the presence of Q1O1, rubisco activity increased, demonstrating a rubisco activase function (Fig. 3b,c). Inclusion of 5% v/v of the crowding agent polyethylene glycol 3,350 (PEG) approximately doubled activase activity (Fig. 3b,c) as observed for the plant Rca system previously^47^.

Due to loose inhibitor binding, form II rubiscos generally do not form stable complexes with RuBP or other physiologically relevant inhibitors, but readily activate in assay buffer^15^,^48^,^49^, which would make an activase unnecessary. Accordingly, no activase has been described for form II rubiscos. Consistent with these considerations, when assaying AfM ER at 20 mM NaHCO_3_ the inhibited complex spontaneously activated to reach full activity after 100 s (Supplementary Fig. 9a). However, when performing rubisco assays at low CO_2_ concentrations (5 mM NaHCO_3_) we observed that the activities of both AfM ECM and ER rapidly converged to a low rate (0.55 s^−1^) suggesting the establishment of an equilibrium of ECM and inactive complexes (Fig. 3d). Assaying either ER or ECM in the presence of Q2O2 resulted in a approximately fivefold increase in linear rubisco activity to achieve a carboxylation rate of 2.7 s^−1^ (Fig. 3d). This is consistent with an active removal of inhibited ER complexes from the equilibrium, which then favours the active ECM form. In contrast to RuBP, AfM bound to the transition state analogue CABP (ECMC) formed stable and inactive complexes (Fig. 3e). Inclusion of Q2O2 in the assay resulted in rapid activation and a rubisco activity equivalent to ECM in the presence of Q2O2 (Fig. 3e). Q2O2 was also functional at high CO_2_ (20 mM NaHCO_3_) as judged by its ability to activate the stably inhibited ECMC complex (Supplementary Fig. 9b). In contrast to Q1O1, activation of form II rubisco by Q2O2 was too fast to resolve the gradual increase in rubisco activity (Fig. 3d,e). Reducing the concentration of Q2O2 resulted in rapid activation of a fraction of active sites, followed by linear rubisco activity (Supplementary Fig. 9c). This observation can be explained by the continual loss of newly formed ECM sites by decarbamylation and/or renewed inhibition (by CABP or RuBP) when activase concentrations are sub-saturating.

The ability of both Q1O1 and Q2O2 to remove the non-physiological tight-binding CABP from the active site of their respective target rubiscos is remarkable, as Rca from spinach is unable to activate CABP-inhibited spinach rubisco^50^.

AfQ2 and Q2O2ΔC444 complexes were unable to activate rubisco, highlighting the importance of the hetero-oligomer in contrast to the homo-oligomeric CbbX and Rca activases (Supplementary Fig. 9d). Q1O1 was specific for AfLS and likewise Q2O2 could only activate AfM (Supplementary Fig. 9e,f). Having established the QO complexes as rubisco activases we then examined their ATPase function.

**Figure 4:**
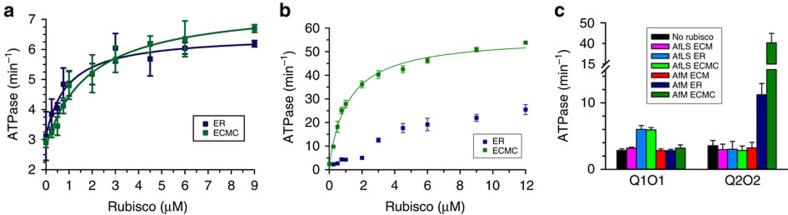
Inhibited rubisco complexes stimulate CbbQO ATPase activity. (**a**,**b**) ATPase activity assays of Q1O1 (**a**) and Q2O2 (**b**) (0.27 μM oligomer) in the presence of varying concentrations of inhibited AfLS (**a**) or AfM (**b**) complexes. (**c**) The ATPase stimulation is isoform specific. ATPase activity of 0.27 μM QO complex was measured in the presence (coloured bars) and absence (black bars) of the indicated rubisco complex (3 μM active sites). Error bars indicate the mean and s.d. of at least three independent experiments.

Both AfQ2 and Q2O2ΔC444 displayed a low ATPase activity of <1 min^−1^ protomer CbbQ^−1^ (Supplementary Fig. 9g). In contrast both Q1O1 and Q2O2 hydrolysed ATP with a specific activity of ∼3 min^−1^ protomer CbbQ^−1^. Therefore, the interaction of the full length CbbO subunit with the CbbQ hexamer induces a conformational change that increases the basal ATP hydrolysis rate of CbbQ. The ATPase activity of both QO systems (but not AfQ2 or Q2O2ΔC444) was specifically stimulated by their respective ER and ECMC complexes in a concentration-dependent manner, resembling the behaviour of CbbX^19^ (Fig. 4a,b, Supplementary Fig. 9g). In contrast, the ATPase activity of Rca is not stimulated by inhibited rubisco complexes^51^.

Activated forms (ECM) of both rubiscos did not result in stimulation (Fig. 4c). The magnitude of the stimulation varied greatly, with saturating concentrations of stably inhibited rubisco increasing Q1O1 and Q2O2 ATPase activity 2- and 20-fold, respectively (Fig. 4a,b). These results demonstrate that the CbbQO complexes specifically recognize inhibited rubisco complexes and that, similar to other AAA+ ATPases^19^,^52^, a productive interaction with their protein substrate results in conformational changes that accelerate ATP hydrolysis and thus assist remodelling.

### The mechanism of QO-mediated rubisco activation is conserved

To probe the mechanism of QO-mediated rubisco activation we purified and characterized a series of amino-acid exchanges in both the rubisco and activase components of the system (Supplementary Figs 10–12). In all cases the fully activated (ECM) rubisco activity of the rubisco mutants tested was at least 70% of the corresponding wild-type activity, indicating catalytic performance was largely unaffected (Supplementary Figs 11d,f and 12d).

**Figure 5:**
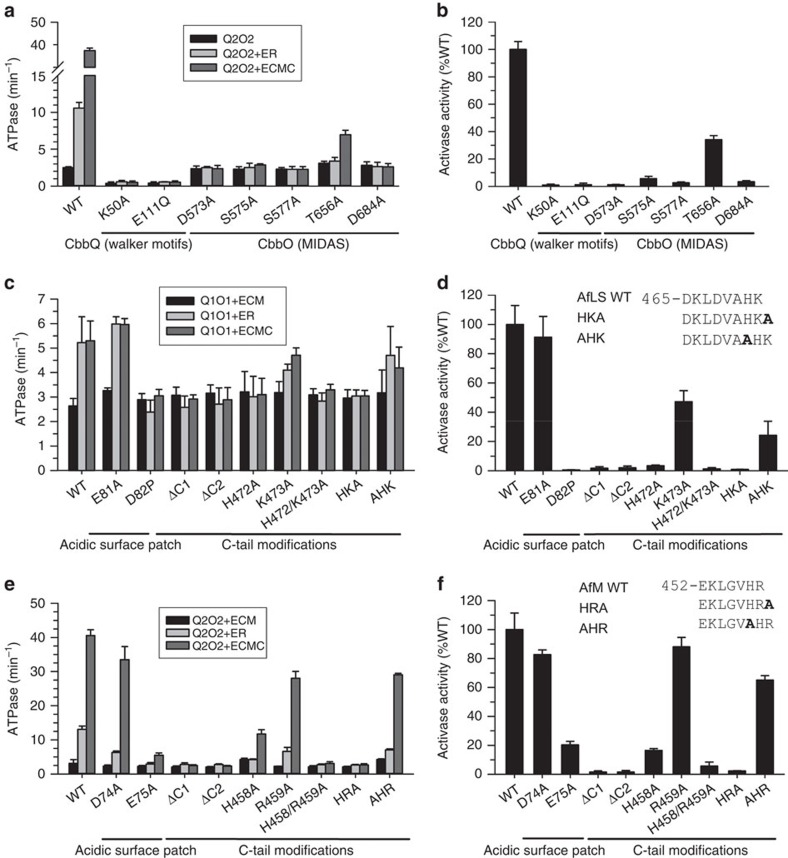
The mechanism of CbbQO mediated rubisco activation is conserved. (**a**–**f**) Identification of residues in Q2O2 and both forms of rubisco that affect activase function and the stimulation of ATPase activity. ATPase activity assays of the indicated QO complexes (0.27 μM oligomer) in the presence and absence of active and inactive rubisco complexes (3 μM active sites) (**a**,**c**,**e**) and normalized rubisco activase activity (**b**,**d**,**f**) carried out using mutated Q2O2 (**a**,**b**), AfLS (**c**,**d**) and AfM (**e**,**f**). Protein concentrations used for activase assays: QO complexes, 0.27 μM oligomer; AfLS, 0.3 μM active sites; AfM, 0.1 μM active sites. Activase assays were performed using ECMC complexes. Error bars indicate the mean and s.d. of at least three independent experiments.

For the AAA+ ATPase AfQ2 we mutated the Walker A and Walker B motifs of the nucleotide-binding domain. Consistent with a motor function of CbbQ, these mutations abolished both ATPase and activase activities of the Q2O2 mutants (Fig. 5a,b).

For CbbX and other AAA+ ATPases the so-called pore-loop 1, which lines the pore of the ring-shaped hexamer, has been shown to be important for remodelling protein substrates^19^,^22^,^23^. This is generally achieved by threading a peptide through the pore. CbbQ has a conserved pore-loop 1 motif, but the aromatic residue attributed to threading in other systems is replaced by a leucine (Supplementary Fig. 10a). A number of predicted AfQ2 pore-loop 1 mutants maintained activase functionality (L79Y, V80A, V80F, W83L and W83F; Supplementary Fig. 10c). The W83L and W83F mutants displayed three and twofold increased basal ATPase activities. Although the D78A substitution resulted in a loss of activase function, a concomitant abolition in ATPase activity makes this result inconclusive (Supplementary Fig. 10b). Analysis of less-conservative amino-acid exchanges in this region was hampered by the insolubility of such mutants.

In both CbbX and Rca pore-loop mutations leading to ATPase-active, but activase-compromised proteins have been identified^19^,^20^. In contrast, our data so far do not support a role for the central pore of the AAA+ hexamer in the mechanism of CbbQO-mediated rubisco activation.

To understand the contribution of the CbbO subunit we mutated all five predicted CbbO MIDAS motif residues in the VWA domain of Q2O2 to alanine (Supplementary Fig. 2). In other systems, such as the integrins, the MIDAS motif mediates protein–protein interactions via a metal ion (usually Mg^2+^), which is co-ordinated by the five conserved residues^40^. All MIDAS mutants tested were successfully purified as the Q2O2 hetero-oligomeric complex (Supplementary Fig. 11a). Consistent with an important role in rubisco–activase interaction all mutants but one (Q2O2(T656A)) were ATPase active, but unable to activate rubisco (Fig. 5a,b). In addition their ATPase activity was no longer stimulated by inhibited AfM (Fig. 5a), which indicates that the mutant activases could no longer sense their substrate.

The MIDAS motif generally interacts with the side chain of an acidic residue on the interacting protein, which provides a sixth co-ordination site for the MIDAS ion^39^,^40^. Following extensive mutagenesis of surface exposed AfLS Glu and Asp residues, we discovered that activation of AfL(D82P)S by Q1O1 was eliminated (Fig. 5d). Inhibited AfL(D82P)S did not stimulate the ATPase activity of Q1O1 (Fig. 5c). Remarkably, when the corresponding residue in AfM (E75) was mutated to alanine, activation of the resultant form II enzyme was reduced by 80% and stimulation of Q2O2 ATPase activity was abolished (Fig. 5e,f). It is striking that the corresponding residue in higher plant large subunits is 89, which is essential for rubisco activation by Rca^26^ and interacts with a helical insertion in the small subdomain of the Rca AAA+ module^20^,^25^,^53^. Mutation to alanine of directly adjacent acidic residues (E81 and D74, respectively) did not affect the enzymes' ability to be activated by their activases (Fig. 5d,f) and underlined the specificity of the interaction.

These results support a model where CbbO functions as an essential substrate adaptor that binds a specific surface-exposed carboxylate group of inhibited rubisco via its VWA domain.

A sequence alignment of rubisco large subunit sequences associated with *cbbQ* and *cbbO* revealed the presence of an HK/R motif at their C terminus not present in other form I and form II sequences (Supplementary Fig. 11b). For both AfLS and AfM deleting one or two residues (ΔC1 and ΔC2), or adding one alanine residue to the C terminus of the large subunit (HKA and HRA) abolished activase function and ATPase stimulation (Fig. 5c–f). In contrast inserting an alanine upstream of the HK/R motif reduced, but did not eliminate the ability of rubisco to be activated. Therefore, the presence and relative position of the C-terminal HK/R motif is critical. Additional analysis showed that the final residue could be substituted without a marked loss of function, whereas the penultimate histidine residue could only be partially replaced by aromatic residues (Fig. 5c–f, Supplementary Fig. 12).

A peptide corresponding to the final 11 residues of the AfM C terminus was found to strongly stimulate the ATPase activity of Q2O2, albeit at much higher concentrations (in the mM range) than required for stimulation by inhibited rubisco complexes (Supplementary Fig. 12e). In contrast an 11-mer including the identified interacting acidic surface residue of AfM (E75) did not result in stimulation. Hence the C-terminal peptide in isolation can interact productively with the CbbQO complex, but additional contacts are required for a high-affinity interaction.

These results show that the CbbQO activases, like CbbX^19^, also function via manipulation of the large subunit C terminus. However, the interaction between the rubisco large subunit C terminus and CbbX is less specific than for CbbQO, since in the former system both the deletion of one C-tail residue or substitution of the only conserved residue was tolerated^19^. The relatively stringent requirements exhibited by CbbQO activation concerning the architecture of the C terminus may indicate that in this instance the C terminus binds to a specific pocket in the CbbQO complex.

## Discussion

In this work we demonstrate the existence of a third class of rubisco activase in chemoautotrophic bacteria and for the first time extend this phenomenon to the distantly related form II rubiscos. Our observations suggest that both sugar-phosphate-mediated inhibition and consequently the requirement for activase proteins is remarkably wide-spread among rubiscos and raises the possibility of universality. If so, then we can expect more convergently evolved rubisco activation systems to be discovered in organisms whose genomes do not encode Rca, CbbX or CbbQO.

*A. ferrooxidans* possesses multiple rubisco-encoding operons, the two studied here and an additional gene cluster encoding carboxysomal gene products, form I rubisco and a third set of *cbbQ* and *cbbO* genes^54^. In *Hydrogenovibrio marinus* it has been convincingly shown that three similar operons are regulated in response to CO_2_ concentrations^55^, and this also appears to be the case in *A. ferrooxidans*^56^. At high CO_2_ concentrations form II rubisco prevails, whereas when CO_2_ is limiting, the form I enzymes are present. On the basis of our biochemical data, Q2O2 function is predicted to be less important at high CO_2_ concentrations, since the ER complex is unstable (Supplementary Fig. 9a). However, it is likely and so far unexplored, that other inhibitors, such as rubisco misfire products^16^, exist in chemoautotrophic bacteria. Some of these may form complexes that resemble ECMC and thus necessitate Q2O2 function at high CO_2_ concentrations.

**Figure 6:**
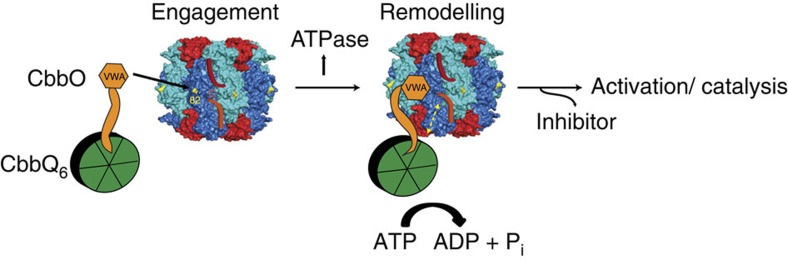
Proposed model of CbbQO function visualized using form I rubisco. CbbQO binds to inhibited rubisco complexes via the acidic surface residue (Asp 82, coloured yellow) of the rubisco large subunit using the MIDAS binding site located on CbbO. The AAA+ hexamer formed by CbbQ may also interact with rubisco directly, possibly via the C terminus of the large subunit (indicated by yellow dashed double arrow). Productive binding, which involves both Asp 82 and the C terminus, results in a stimulation of ATPase activity. This provides the energy used to remodel the inhibited rubisco active site allowing release of the inhibitor. The CbbQO model is not drawn to scale. The form I rubisco model is from *Halothiobacillus neapolitanus* (pdb:1SVD).

In spite of the different quaternary structure and low sequence identity (33%) between form I and form II rubisco, the similar mutant phenotypes demonstrate that the mechanism of QO-mediated rubisco activation is conserved. Our results support an initial model where a CbbQ_6_O_1_ complex interacts with the inhibited rubisco via the MIDAS motif of the VWA domain of CbbO, by engaging the identified acidic surface residue Glu 75 (form II) or Asp 82 (form I) (Fig. 6). The residue in plant rubisco that corresponds to the identified acidic surface residue, Pro 89, has been demonstrated to be necessary for activation by Rca^26^, which indicates mechanistic similarities in rubisco activation. As observed for CbbX, a productive interaction between the activase and rubisco results in a stimulation of ATPase activity (Fig. 4), which powers conformational remodelling of the inhibited enzyme. As a result the active site opens and the inhibitory sugar is released. As in CbbX, manipulation of the C terminus of the large subunit is necessary (Fig. 5), but so far no evidence for an involvement of the CbbQ pore has been obtained (Supplementary Fig. 10). It remains to be established whether direct CbbQ–rubisco contacts are involved in the mechanism, which would allow direct force transmission from the ATPase to the substrate and permit a passive, binding role for CbbO. Alternatively the mechanical force leading to active site disruption could be transmitted through the CbbO linker to the VWA domain. We favour a mechanism similar to that proposed for the ribosome maturation protein Rea1 or midasin^57^. Rea1, the largest yeast protein, possesses six AAA+ modules connected to one VWA domain at its C terminus via a long linker and thus resembles the organization of the CbbQO hetero-oligomer. In comparative genomics, it has been noted that fusion proteins of different domains can predict the subunit organization of related protein complexes^58^. Since the AAA+ module of Rea1 has been placed in the same group as CbbQ in two independent classification studies^59^,^60^, it may predict the subunit organization of CbbQO. Rea1 removes the preribosomal factor Rsa4 and the Rix1-subcomplex from the pre-60S subunit. The Rea1 VWA domain binds Rsa4 via its MIDAS motif, whereas the AAA moiety is bound to the Rix1-subcomplex. A force generated by ATP hydrolysis then disrupts the complex, leading to release of Rsa4, the Rix1-subcomplex and Rea1 from the pre-60S particle^57^. It will be interesting to see whether disruption of the rubisco active site by CbbQO proceeds via such a mechanism.

Since CbbQO activation involves conserved contact points that have been shown to be important for activation by Rca and CbbX respectively (residue 89 and the C terminus), it is tempting to speculate that the active site of the rubisco large subunit is subjected to a very similar force application during activation by the three different systems. Since rubisco large subunit secondary structure is highly conserved^9^, the conformational changes that lead to an opening of the inhibited active site are likely to be similar despite large primary sequence divergence. In contrast, the detailed mechanism of force generation and propagation by the three activases appears to be dissimilar, as could be expected for convergently evolved systems.

The MoxR AAA+ family is poorly studied, but in most cases is associated with a VWA-domain-containing protein^38^. On the basis of our results we hypothesize that the association of a AAA+ hexamer with one VWA protein subunit will be a general theme. This hetero-oligomeric arrangement requires an asymmetric binding mode during complex assembly. It can be envisaged that interaction of a CbbO monomer with a AAA+ hexamer leads to a conformational change that prevents the binding of additional CbbO subunits.

We predict that in similar systems the VWA protein will bind a target protein via its MIDAS motif, and that the energy of ATP hydrolysis will then be employed to conformationally remodel the substrate. Consistent with these ideas the VWA protein ViaA stimulates the ATPase activity of its associated MoxR AAA+ protein RavA, suggesting an interaction^61^. Likewise the viral MoxR AAA+ protein p618 has been shown to interact with the associated VWA protein p892 (ref. 62).

Early work by Hayashi *et al.*^31^,^32^ suggested increased rubisco activity in *E. coli* lysates co-expressing rubisco and associated CbbQ and CbbO proteins. This increased activity may possibly be explained by removal of so far unidentified inhibitors present in the *E. coli* cytoplasm. However, the increased activity was reported for the CbbQ and CbbO proteins expressed separately^32^ and specificity was not observed (for example, both CbbQ1 and CbbQ2 co-expression led to increased form I and form II rubisco activity^31^). In contrast our *in vitro* data show the entire CbbQO complex is required for functionality. So far, we have not been able to identify CbbO proteins that express solubly in *E. coli* in the absence of CbbQ.

In summary our work illuminates another facet of the evolutionary innovations developed by organisms to overcome the well-documented short-comings of rubisco. A thorough mechanistic understanding of these processes will allow engineering strategies towards optimized CO_2_ fixation to be implemented with consequences in fields such as agriculture, bioenergy and CO_2_ sequestration.

## Methods

### Plasmids

*A. ferrooxidans* ATCC 23270 (ref. 63) genes were amplified from genomic DNA obtained from the ATCC. The *cbbL* (AFE_3051) and *cbbS* (AFE_3052) genes are positioned in tandem and were amplified together and cloned between the NdeI–HindIII sites of the vector pET30b to yield pET30b*AfcbbLS*. The form II rubisco *cbbM* (AFE_2155), activase genes *cbbQ1* (AFE_3053), and *cbbQ2* (AFE_2156), were cloned between the SacII/HindIII sites of the vector pHue allowing cleavage of His_6_–ubiquitin fusions at their native N termini^46^. This resulted in plasmids pHue*AfcbbM*, pHue*AfcbbQ1* and pHue*AfcbbQ2* respectively. pHue*RrcbbM* and pHue*RpcbbM* encoding form II rubisco of *Rhodospirillum rubrum* (RrM) and *Rhodopseudomonas palustris* (RpM) were constructed similarly, but the former used pTrcrbcM^64^ as template and was cloned between the BamHI/HindIII sites of pHue, and *RpcbbM* was synthesized by Genscript. *CbbO1* (AFE_3054) and *cbbO2* (AFE_2157) were first amplified and cloned into the pHue vector using SacII and HindIII restriction sites followed by positioning the XbaI-HindIII fragments into the pBAD33 vector^65^ to give pBAD33*UbAfcbbO1* and pBAD33*UbAfcbbO2*. pBAD33*Ub-*HA*-AfcbbO2* and pBAD33*Ub-*FLAG*-AfcbbO1* were constructed in the same way, but using pHueHA and pHueFLAG instead of pHue (these vectors have sequences encoding the HA and the FLAG-epitopes inserted 5′ of the SacII site, and 3′ of the protease cleavage site resulting in N-terminal epitope tags). The QuikChange protocol (Stratagene) was used to introduce point mutations, deletions or insertions into the expression plasmids as desired. The plasmids and primers used are listed in Supplementary Table 1 and 2, respectively. All protein-encoding sequences were verified by DNA sequencing.

### Protein expression and purification

All protein concentrations were quantified using the Bradford assay with bovine serum albumin as the standard. Immunoblotting used monoclonal mouse anti-FLAG M2 (Sigma Aldrich, F1804) and anti-HA (protein tech, 66006) antibodies at 1:1,000 dilution.

Plasmids encoding His_6_–ubiquitin fusion proteins (AfM and mutants, RpM, RrM, AfQ1 and AfQ2) were transformed into *E. coli* BL21 (DE3) cells and grown in Luria–Bertani (LB) medium at 37 °C followed by induction with 0.5 mM isopropyl ß-D-1-thiogalactopyranoside (IPTG) at 23 °C. The collected cells were lysed in buffer A (20 mM Tris-HCl, pH 8.0, 50 mM NaCl) containing 10 mM imidazole and 0.3 mg ml^−1^ lysozyme for 30 min on ice. Cells were disrupted by ultrasonication after addition of 1 mM phenylmethanesulfonyl fluoride. The supernatant obtained from centrifugation (40,000 × *g*, 45 min, 4 °C) was applied to Ni^2+^-nitrilotriacetic acid resin (Pierce) followed by cleavage of the ubiquitin moiety to yield the native N terminus, as described previously^46^. Any precipitated protein was removed at this stage by centrifugation. The protein solution was applied to a pre-equilibrated Mono Q 10/100 GL column, and eluted with a linear salt gradient to 0.5 M NaCl. Fractions containing the protein of interest were combined, concentrated and applied to a Superdex 200 gel-filtration column equilibrated with buffer A. The purest protein fractions (>95% pure as judged by SDS–PAGE) were concentrated, supplemented with 5% glycerol, flash-frozen in liquid nitrogen and stored at −80 °C.

*E. coli* BL21 cells transformed with pET30b*AfcbbLS* encoding wild-type and mutant proteins were grown and induced as described above. The cells were resuspended in buffer A, incubated with lysozyme and lysed by ultrasonication. The soluble supernatant was applied to a Source30Q column (GE Healthcare) pre-equilibrated with buffer A and proteins were eluted with a linear salt gradient to 0.5 M NaCl. Fractions containing AfLS were combined, concentrated and applied to Superdex 200 16/60 size-exclusion column. The purest fractions were pooled, concentrated, supplemented with 5% v/v glycerol, flash-frozen in liquid nitrogen and stored at −80 °C.

AfCbbQ1 and AfCbbO1 were co-expressed in *E. coli* and co-purified as Q1O1 His_6_–ubiquitin fusion proteins. The cells harbouring both pHue*AfcbbQ1* and pBAD33*UbAfcbbO1* were grown in LB medium and induced with both 0.5 mM IPTG and 0.2% w/v L-arabinose at 23 °C. Soluble lysate was produced and Ni-NTA affinity chromatography was performed as described above. After overnight cleavage of the ubiquitin moiety at 23 °C, the soluble proteins were applied onto a MonoQ column followed by gel-filtration using a Superdex 200 column. AfCbbQ2 and AfCbbO2 (and all Q2O2 mutants) were also co-expressed and co-purified like Q1O1 protein with the addition of 5 mM MgCl_2_ and 5% glycerol in the purification buffers used in Ni-NTA agarose and anion-exchange columns. Purified proteins were concentrated to∼5 mg ml^−1^, 5% glycerol was added, flash-frozen in liquid nitrogen and stored at −80 °C.

*Rhodobacter sphaeroides* form I rubisco (RsLS) was purified as described^19^.

### Determination of native molecular weight

Analytical gel filtration was performed using a Superdex 200 PC3.2/30 column using buffer A as the eluant. For glycerol gradient sedimentation analysis, 10 ml 5–30% glycerol gradients in buffer A were overlaid with 100 μl of protein solution in buffer A (the concentration of each complex was 1 mg ml^−1^). The gradients were centrifuged (35,000 r.p.m., 16 h, 4 °C) using a SW 41 Ti rotor (Beckmann Coulter). Five hundred microlitres fractions were collected and analysed by SDS–PAGE and silver staining. Standards for both gel filtration and sedimentation analysis were obtained from the high-molecular-weight gel filtration calibration kit (GE healthcare). Native molecular weight was calculated using the equation *M*=4,205 (S*R*_s_), where S is in Svedberg units and *R*_s_ in nanometers^43^.

### Enzymatic assays

All ATPase activity and rubisco activation assays were performed spectrophotometrically at 25 °C. ATPase activity was assayed using a coupled enzymatic assay that monitored the oxidation of NADH as described previously^19^,^66^. Rubisco activity was assayed using the spectrophotometric assay essentially as described^67^ with minor modifications. Briefly, the measurements were carried out in 100-μl reactions containing 100 mM Tricine-NaOH pH8.0, 5 mM MgCl_2_, coupling enzymes^67^, 0.5 mM NADH, 10 mM phosphocreatine, 5 or 20 mM NaHCO_3_, 1 mM DTT/ATP/RuBP, 0.27 μM CbbQO oligomer, and ECM, ER and ECMC as stated in the figure legends. Ribulose-1,5-bisphosphate was synthesized enzymatically from ribose-5-phosphate^68^ and purified using anion-exchange chromatography^69^. ECM was obtained by incubating rubisco (20–100 μM active sites depending on desired final concentration) in buffer A supplemented with 40 mM NaHCO_3_ and 10 mM MgCl_2_ for 10 min at 25 °C, while ECMC was prepared by further addition of a fourfold molar excess of carboxypentitol bisphosphate (a 1:1 mixture of the tight-binding inhibitor CABP and the loose-binding stereoisomer carboxyribitol-1,5-bisphosphate) to the ECM complex for 10 min. The ER complex was obtained by incubating rubisco in buffer A with 4 mM EDTA for 10 min and then adding 0.8 mM RuBP. Peptides (>95% purity) were synthesized by the Peptide Synthesis Core Facility of the School of Biological Sciences, NTU.

Relative activase activities were determined by measuring the rubisco activity of ECMC complexes exposed to activase 1 min after assay initiation.

### Electron microscopy

Purified proteins at concentrations indicated in the figure legends in 20 mM Tris-HCl pH8.0, 50 mM NaCl were applied to a carbon-coated TEM grid and stained with 2% (w/v) uranyl acetate. Micrographs were recorded on a FEI T12 transmission electron microscope equipped with a 4 K CCD camera (FEI) at a magnification of × 66,350 resulting in a pixel size of 2.11 Å per pixel under low-dose conditions. Single particles were selected and processed with the EMAN2 image-processing package^70^. Particles were CTF corrected with EMAN2 before processing.

Particles were manually picked for the AfQ2 data set (defocus range 0.8–1.8 μm) because of the preferred orientation of particles on the grid (with top views dominating). A total of 958 particles were used for reference-free unbiased 2D classifications and 3D reconstruction. Unbiased 2D classification and initial models clearly suggested a six-fold symmetry similar to other known hexameric AAA proteins. The highest quality initial model (based on EMAN2 criterion) was used for further refinement to obtain a 3D EM map of AfQ2 at 23 Å resolution using gold standard criteria (0.143 criterion) with an imposed six-fold symmetry.

A data set of Q2O2ΔC444 (defocus range 0.6–1.6 μm) containing 12,486 particles was used for reference-free unbiased 2D classifications. Selected 2D classes showed an additional density of O2 radiating from one of the subunits of the AfQ2 hexamer when compared with 2D classes of AfQ2 alone.

Figure 6 was prepared using Pymol (www.pymol.org).

## Additional information

** Accession codes:** The EM density map of AfQ2 has been deposited in the Electron Microscopy Data Bank with accession number EMD-6477.

**How to cite this article:** Tsai, Y.-C.C. *et al.* Identification and characterization of multiple rubisco activases in chemoautotrophic bacteria. *Nat. Commun.* 6:8883 doi: 10.1038/ncomms9883 (2015).

## Supplementary Material

Supplementary Information Supplementary Figures 1-12, Supplementary Tables 1-2 and Supplementary References

## Figures and Tables

**Table 1:** Native molecular weight determination of rubisco and CbbQO complexes.

**Protein complex**	**Svedberg coefficient (S)**	**Stokes radius (nm)**	**Native molecular weight (kDa)**	**Predicted molecular weight (Da)**	**Ratio measured: predicted.**
RsLS	18.2	7.0	536	550,339.2 (L_8_S_8_)	0.97 : 1
AfLS	17.2	6.9	499	530,056 (L_8_S_8_)	0.94 : 1
RrM	6.1	4.5	115	100,964 (L_2_)	1.14 : 1
AfM	12.0	6.2	313	302,298 (L_6_)	1.03 : 1
RpM	12.2	6.0	308	324,330 (L_6_)	0.95 : 1
AfQ2	7.6	5.4	173	181,029.6 (Q_6_)	0.96 : 1
Q1O1	10.3	6.1	264	267,253.4 (Q_6_O_1_)	0.99 : 1
Q2O2	11.1	6.1	285	266,437 (Q_6_O_1_)	1.07 : 1
Q2O2ΔC444	8.5	6.1	218	231,122.3 (Q_6_O_1_)	0.94 : 1

## References

[b1] Lin M. T., Occhialini A., Andralojc P. J., Parry M. A. & Hanson M. R. A faster Rubisco with potential to increase photosynthesis in crops. Nature 513, 547–550 (2014). 25231869 10.1038/nature13776 PMC4176977

[b2] Parry M. A. *et al.* Rubisco activity and regulation as targets for crop improvement. J. Exp. Bot. 64, 717–730 (2013). 23162118 10.1093/jxb/ers336

[b3] Zhu X. G., Long S. P. & Ort D. R. Improving photosynthetic efficiency for greater yield. Annu. Rev. Plant Biol. 61, 235–261 (2010). 20192734 10.1146/annurev-arplant-042809-112206

[b4] Whitney S. M., Baldet P., Hudson G. S. & Andrews T. J. Form I Rubiscos from non-green algae are expressed abundantly but not assembled in tobacco chloroplasts. Plant J. 26, 535–547 (2001). 11439139 10.1046/j.1365-313x.2001.01056.x

[b5] Whitney S. M., Houtz R. L. & Alonso H. Advancing our understanding and capacity to engineer nature's CO(2)-sequestering enzyme, rubisco. Plant Physiol. 155, 27–35 (2011). 20974895 10.1104/pp.110.164814 PMC3075749

[b6] Whitney S. M., Birch R., Kelso C., Beck J. L. & Kapralov M. V. Improving recombinant Rubisco biogenesis, plant photosynthesis and growth by coexpressing its ancillary RAF1 chaperone. Proc. Natl Acad. Sci. USA 112, 3564–3569 (2015). 25733857 10.1073/pnas.1420536112 PMC4371954

[b7] Mueller-Cajar O., Stotz M. & Bracher A. Maintaining photosynthetic CO2 fixation via protein remodelling: the Rubisco activases. Photosynth. Res. 119, 191–201 (2014). 23543331 10.1007/s11120-013-9819-0

[b8] Hauser T., Popilka L., Hartl F. U. & Hayer-Hartl M. Role of auxiliary proteins in Rubisco biogenesis and function. Nat. Plants 1, 15065 (2015). 10.1038/nplants.2015.65 27250005

[b9] Andersson I. & Backlund A. Structure and function of Rubisco. Plant Physiol. Biochem. 46, 275–291 (2008). 18294858 10.1016/j.plaphy.2008.01.001

[b10] Satagopan S., Chan S., Perry L. J. & Tabita F. R. Structure-function studies with the unique hexameric form II ribulose-1,5-bisphosphate carboxylase/oxygenase (Rubisco) from Rhodopseudomonas palustris. J. Biol. Chem. 289, 21433–21450 (2014). 24942737 10.1074/jbc.M114.578625 PMC4118107

[b11] Badger M. R. & Bek E. J. Multiple Rubisco forms in proteobacteria: their functional significance in relation to CO2 acquisition by the CBB cycle. J. Exp. Bot. 59, 1525–1541 (2008). 18245799 10.1093/jxb/erm297

[b12] Bauwe H., Hagemann M. & Fernie A. R. Photorespiration: players, partners and origin. Trends Plant Sci. 15, 330–336 (2010). 20403720 10.1016/j.tplants.2010.03.006

[b13] Tcherkez G. G. B., Farquhar G. D. & Andrews T. J. Despite slow catalysis and confused substrate specificity, all ribulose bisphosphate carboxylases may be nearly perfectly optimized. Proc. Natl Acad. Sci. USA 103, 7246–7251 (2006). 16641091 10.1073/pnas.0600605103 PMC1464328

[b14] Pearce F. G. & Andrews T. J. The relationship between side reactions and slow inhibition of ribulose-bisphosphate carboxylase revealed by a loop 6 mutant of the tobacco enzyme. J. Biol. Chem. 278, 32526–32536 (2003). 12783874 10.1074/jbc.M305493200

[b15] Pearce F. G. Catalytic by-product formation and ligand binding by ribulose bisphosphate carboxylases from different phylogenies. Biochem. J. 399, 525–534 (2006). 16822231 10.1042/BJ20060430 PMC1615894

[b16] Parry M. A. J., Keys A. J., Madgwick P. J., Carmo-Silva A. E. & Andralojc P. J. Rubisco regulation: a role for inhibitors. J. Exp. Bot. 59, 1569–1580 (2008). 18436543 10.1093/jxb/ern084

[b17] Cleland W. W., Andrews T. J., Gutteridge S., Hartman F. C. & Lorimer G. H. Mechanism of Rubisco—the carbamate as general base. Chem. Rev. 98, 549–561 (1998). 11848907 10.1021/cr970010r

[b18] Portis A. R. Rubisco activase—Rubisco's catalytic chaperone. Photosynth. Res. 75, 11–27 (2003). 16245090 10.1023/A:1022458108678

[b19] Mueller-Cajar O. *et al.* Structure and function of the AAA+ protein CbbX, a red-type Rubisco activase. Nature 479, 194–199 (2011). 22048315 10.1038/nature10568

[b20] Stotz M. *et al.* Structure of green-type Rubisco activase from tobacco. Nat. Struct. Mol. Biol. 18, 1366–1370 (2011). 22056769 10.1038/nsmb.2171

[b21] Salvucci M. E., Portis A. R. & Ogren W. L. A soluble chloroplast protein catalyzes ribulosebisphosphate carboxylase oxygenase activation in vivo. Photosynth. Res. 7, 193–201 (1985). 24443088 10.1007/BF00037012

[b22] Sauer R. T. & Baker T. A. AAA+ Proteases: ATP-fueled machines of protein destruction. Annu. Rev. Biochem. 80, 587–612 (2011). 21469952 10.1146/annurev-biochem-060408-172623

[b23] Weibezahn J. *et al.* Thermotolerance requires refolding of aggregated proteins by substrate translocation through the central pore of ClpB. Cell 119, 653–665 (2004). 15550247 10.1016/j.cell.2004.11.027

[b24] Scales J. C., Parry M. A. & Salvucci M. E. A non-radioactive method for measuring Rubisco activase activity in the presence of variable ATP: ADP ratios, including modifications for measuring the activity and activation state of Rubisco. Photosynth. Res. 119, 355–365 (2014). 24390640 10.1007/s11120-013-9964-5 PMC3923112

[b25] Henderson J. N., Kuriata A. M., Fromme R., Salvucci M. E. & Wachter R. M. Atomic resolution X-ray structure of the substrate recognition domain of higher plant ribulose-bisphosphate carboxylase/oxygenase (rubisco) activase. J. Biol. Chem. 286, 35683–35688 (2011). 21880724 10.1074/jbc.C111.289595 PMC3195603

[b26] Larson E. M., O'Brien C. M., Zhu G., Spreitzer R. J. & Portis A. R. Jr. Specificity for activase is changed by a Pro-89 to Arg substitution in the large subunit of ribulose-1,5-bisphosphate carboxylase/oxygenase. J. Biol. Chem. 272, 17033–17037 (1997). 9202018 10.1074/jbc.272.27.17033

[b27] Ott C. M., Smith B. D., Portis A. R. Jr. & Spreitzer R. J. Activase region on chloroplast ribulose-1,5-bisphosphate carboxylase/oxygenase. J. Biol. Chem. 275, 26241–26244 (2000). 10858441 10.1074/jbc.M004580200

[b28] Andralojc P. J. *et al.* 2-Carboxy-D-arabinitol 1-phosphate (CA1P) phosphatase: evidence for a wider role in plant Rubisco regulation. Biochem. J. 442, 733–742 (2012). 22132794 10.1042/BJ20111443

[b29] Bracher A., Sharma A., Starling-Windhof A., Hartl F. U. & Hayer-Hartl M. Degradation of potent Rubisco inhibitor by selective sugar phosphatase. Nat. Plants 1, 14002 (2015). 10.1038/nplants.2014.2 27246049

[b30] Swan B. K. *et al.* Potential for chemolithoautotrophy among ubiquitous bacteria lineages in the dark ocean. Science 333, 1296–1300 (2011). 21885783 10.1126/science.1203690

[b31] Hayashi N. R., Arai H., Kodama T. & Igarashi Y. The cbbQ genes, located downstream of the form I and form II RubisCO genes, affect the activity of both RubisCOs. Biochem. Biophys. Res. Commun. 265, 177–183 (1999). 10548510 10.1006/bbrc.1999.1103

[b32] Hayashi N. R., Arai H., Kodama T. & Igarashi Y. The novel genes, cbbQ and cbbO, located downstream from the RubisCO genes of Pseudomonas hydrogenothermophila, affect the conformational states and activity of RubisCO. Biochem. Biophys. Res. Commun. 241, 565–569 (1997). 9425311 10.1006/bbrc.1997.7853

[b33] Schwedock J. *et al.* Characterization and expression of genes from the RubisCO gene cluster of the chemoautotrophic symbiont of Solemya velum: cbbLSQO. Arch. Microbiol. 182, 18–29 (2004). 15316720 10.1007/s00203-004-0689-x

[b34] Guadalupe-Medina V. *et al.* Carbon dioxide fixation by Calvin-Cycle enzymes improves ethanol yield in yeast. Biotechnol. Biofuels 6, 125 (2013). 23987569 10.1186/1754-6834-6-125 PMC3766054

[b35] Bohnke S. & Perner M. A function-based screen for seeking RubisCO active clones from metagenomes: novel enzymes influencing RubisCO activity. ISME J. 9, 735–745 (2015). 25203835 10.1038/ismej.2014.163 PMC4331584

[b36] Wong K. S. & Houry W. A. Novel structural and functional insights into the MoxR family of AAA+ ATPases. J. Struct. Biol. 179, 211–221 (2012). 22491058 10.1016/j.jsb.2012.03.010

[b37] El Bakkouri M. *et al.* Structure of RavA MoxR AAA plus protein reveals the design principles of a molecular cage modulating the inducible lysine decarboxylase activity. Proc. Natl Acad. Sci. USA 107, 22499–22504 (2010). 21148420 10.1073/pnas.1009092107 PMC3012504

[b38] Snider J. & Houry W. A. MoxR AAA+ ATPases: A novel family of molecular chaperones? J. Struct. Biol. 156, 200–209 (2006). 16677824 10.1016/j.jsb.2006.02.009

[b39] Whittaker C. A. & Hynes R. O. Distribution and evolution of von Willebrand/integrin a domains: Widely dispersed adhesion and elsewhere. Mol. Biol. Cell 13, 3369–3387 (2002). 12388743 10.1091/mbc.E02-05-0259 PMC129952

[b40] Xiong J. P. *et al.* Crystal structure of the extracellular segment of integrin alpha V beta 3 in complex with an Arg-Gly-Asp ligand. Science 296, 151–155 (2002). 11884718 10.1126/science.1069040

[b41] Santelli E., Bankston L. A., Leppla S. H. & Liddington R. C. Crystal structure of a complex between anthrax toxin and its host cell receptor. Nature 430, 905–908 (2004). 15243628 10.1038/nature02763

[b42] Siegel L. M. & Monty K. J. Determination of molecular weights and frictional ratios of proteins in impure systems by use of gel filtration and density gradient centrifugation. Biochim. Biophys. Acta 112, 346–362 (1966). 5329026 10.1016/0926-6585(66)90333-5

[b43] Erickson H. P. Size and shape of protein molecules at the nanometer level determined by sedimentation, gel filtration, and electron microscopy. Biol. Proced. Online 11, 32–51 (2009). 19495910 10.1007/s12575-009-9008-x PMC3055910

[b44] Pierce J., Tolbert N. E. & Barker R. Interaction of ribulosebisphosphate carboxylase-oxygenase with transition-state analogs. Biochemistry 19, 934–942 (1980). 7356969 10.1021/bi00546a018

[b45] Alonso H., Blayney M. J., Beck J. L. & Whitney S. M. Substrate-induced assembly of Methanococcoides burtonii D-ribulose-1,5-bisphosphate carboxylase/oxygenase dimers into decamers. J. Biol. Chem. 284, 33876–33882 (2009). 19837658 10.1074/jbc.M109.050989 PMC2797158

[b46] Catanzariti A.-M., Soboleva T. A., Jans D. A., Board P. G. & Baker R. T. An efficient system for high-level expression and easy purification of authentic recombinant proteins. Protein Sci. 13, 1331–1339 (2004). 15096636 10.1110/ps.04618904 PMC2286746

[b47] Salvucci M. E. Subunit interactions of rubisco activase - polyethylene-glycol promotes self-association, stimulates atpase and activation activities, and enhances interactions with rubisco. Arch. Biochem. Biophys. 298, 688–696 (1992). 1416997 10.1016/0003-9861(92)90467-b

[b48] Jordan D. B. & Chollet R. Inhibition of ribulose bisphosphate carboxylase by substrate ribulose 1,5-bisphosphate. J.Biol.Chem. 258, 13752–13758 (1983). 6417133

[b49] Hernandez J. M., Baker S. H., Lorbach S. C., Shively J. M. & Tabita F. R. Deduced amino acid sequence, functional expression, and unique enzymatic properties of the form I and form II ribulose bisphosphate carboxylase oxygenase from the chemoautotrophic bacterium Thiobacillus denitrificans. J. Bacteriol. 178, 347–356 (1996). 8550452 10.1128/jb.178.2.347-356.1996 PMC177664

[b50] Robinson S. P. & Portis A. R. Release of the nocturnal inhibitor, carboxyarabinitol-1-phosphate, from ribulose bisphosphate carboxylase oxygenase by rubisco activase. FEBS Lett. 233, 413–416 (1988).

[b51] Robinson S. P. & Portis A. R. Adenosine-triphosphate hydrolysis by purified rubisco activase. Arch. Biochem. Biophys. 268, 93–99 (1989). 2912385 10.1016/0003-9861(89)90568-7

[b52] Zhang X. & Wigley D. B. The 'glutamate switch' provides a link between ATPase activity and ligand binding in AAA+ proteins. Nat. Struct. Mol. Biol. 15, 1223–1227 (2008). 18849995 10.1038/nsmb.1501 PMC2806578

[b53] Li C. H., Salvucci M. E. & Portis A. R. Two residues of rubisco activase involved in recognition of the rubisco substrate. J. Biol. Chem. 280, 24864–24869 (2005). 15866868 10.1074/jbc.M503547200

[b54] Heinhorst S. *et al.* Two Copies of form I RuBisCO genes in Acidithiobacillus ferrooxidans ATCC 23270. Curr. Microbiol. 45, 115–117 (2002). 12070689 10.1007/s00284-001-0094-5

[b55] Yoshizawa Y., Toyoda K., Arai H., Ishii M. & Igarashi Y. CO2-responsive expression and gene organization of three ribulose-1,5-bisphosphate carboxylase/oxygenase enzymes and carboxysomes in Hydrogenovibrio marinus strain MH-110. J. Bacteriol. 186, 5685–5691 (2004). 15317772 10.1128/JB.186.17.5685-5691.2004 PMC516815

[b56] Esparza M., Bowien B., Jedlicki E. & Holmes D. S. Gene organization and CO(2)-responsive expression of four cbb operons in the biomining bacterium Acidithiobacillus ferrooxidans. Adv. Mater. Res 71-73, 207–210 (2009).

[b57] Ulbrich C. *et al.* Mechanochemical removal of ribosome biogenesis factors from nascent 60S ribosomal subunits. Cell 138, 911–922 (2009). 19737519 10.1016/j.cell.2009.06.045

[b58] Enright A. J., Iliopoulos I., Kyrpides N. C. & Ouzounis C. A. Protein interaction maps for complete genomes based on gene fusion events. Nature 402, 86–90 (1999). 10573422 10.1038/47056

[b59] Ammelburg M., Frickey T. & Lupas A. N. Classification of AAA+ proteins. J. Struct. Biol. 156, 2–11 (2006). 16828312 10.1016/j.jsb.2006.05.002

[b60] Iyer L. M., Leipe D. D., Koonin E. V. & Aravind L. Evolutionary history and higher order classification of AAA+ ATPases. J. Struct. Biol. 146, 11–31 (2004). 15037234 10.1016/j.jsb.2003.10.010

[b61] Snider J. *et al.* Formation of a distinctive complex between the inducible bacterial lysine decarboxylase and a novel AAA+ ATPase. J. Biol. Chem. 281, 1532–1546 (2006). 16301313 10.1074/jbc.M511172200

[b62] Scheele U. *et al.* Chaperone role for proteins p618 and p892 in the extracellular tail development of Acidianus two-tailed virus. J. Virol. 85, 4812–4821 (2011). 21367903 10.1128/JVI.00072-11 PMC3126172

[b63] Valdes J. *et al.* Acidithiobacillus ferrooxidans metabolism: from genome sequence to industrial applications. BMC Genomics 9, 597 (2008). 19077236 10.1186/1471-2164-9-597 PMC2621215

[b64] Mueller-Cajar O., Morell M. & Whitney S. M. Directed evolution of rubisco in Escherichia coli reveals a specificity-determining hydrogen bond in the form II enzyme. Biochemistry 46, 14067–14074 (2007). 18004873 10.1021/bi700820a

[b65] Guzman L. M., Belin D., Carson M. J. & Beckwith J. Tight regulation, modulation, and high-level expression by vectors containing the arabinose P-Bad promoter. J. Bacteriol. 177, 4121–4130 (1995). 7608087 10.1128/jb.177.14.4121-4130.1995 PMC177145

[b66] Kreuzer K. N. & Jongeneel C. V. Escherichia coli phage-T4 topoisomerase. Methods Enzymol. 100, 144–160 (1983). 6312256 10.1016/0076-6879(83)00051-8

[b67] Kubien D. S., Brown C. M. & Kane H. J. Quantifying the amount and activity of Rubisco in leaves. Methods Mol. Biol. 684, 349–362 (2011). 20960142 10.1007/978-1-60761-925-3_27

[b68] Horecker B. L., Hurwitz J. & Weissbach A. Ribulose diphosphate. Biochem. Prep. 6, 83–90 (1958).

[b69] Kane H. J., Wilkin J. M., Portis A. R. & Andrews T. J. Potent inhibition of ribulose-bisphosphate carboxylase by an oxidized impurity in ribulose-1,5-bisphosphate. Plant Physiol. 117, 1059–1069 (1998). 9662549 10.1104/pp.117.3.1059 PMC34922

[b70] Tang G. *et al.* EMAN2: an extensible image processing suite for electron microscopy. J. Struct. Biol. 157, 38–46 (2007). 16859925 10.1016/j.jsb.2006.05.009

