# Supplementary material for: Identification and characterization of multiple rubisco activases in chemoautotrophic bacteria
Source: Nat Commun. 2015 Nov 16;6:8883. doi: 10.1038/ncomms9883 (PMC4660213; doi:10.1038/ncomms9883)
Supplement: Supplementary Information — Supplementary Figures 1-12, Supplementary Tables 1-2 and Supplementary References [file ncomms9883-s1.pdf]

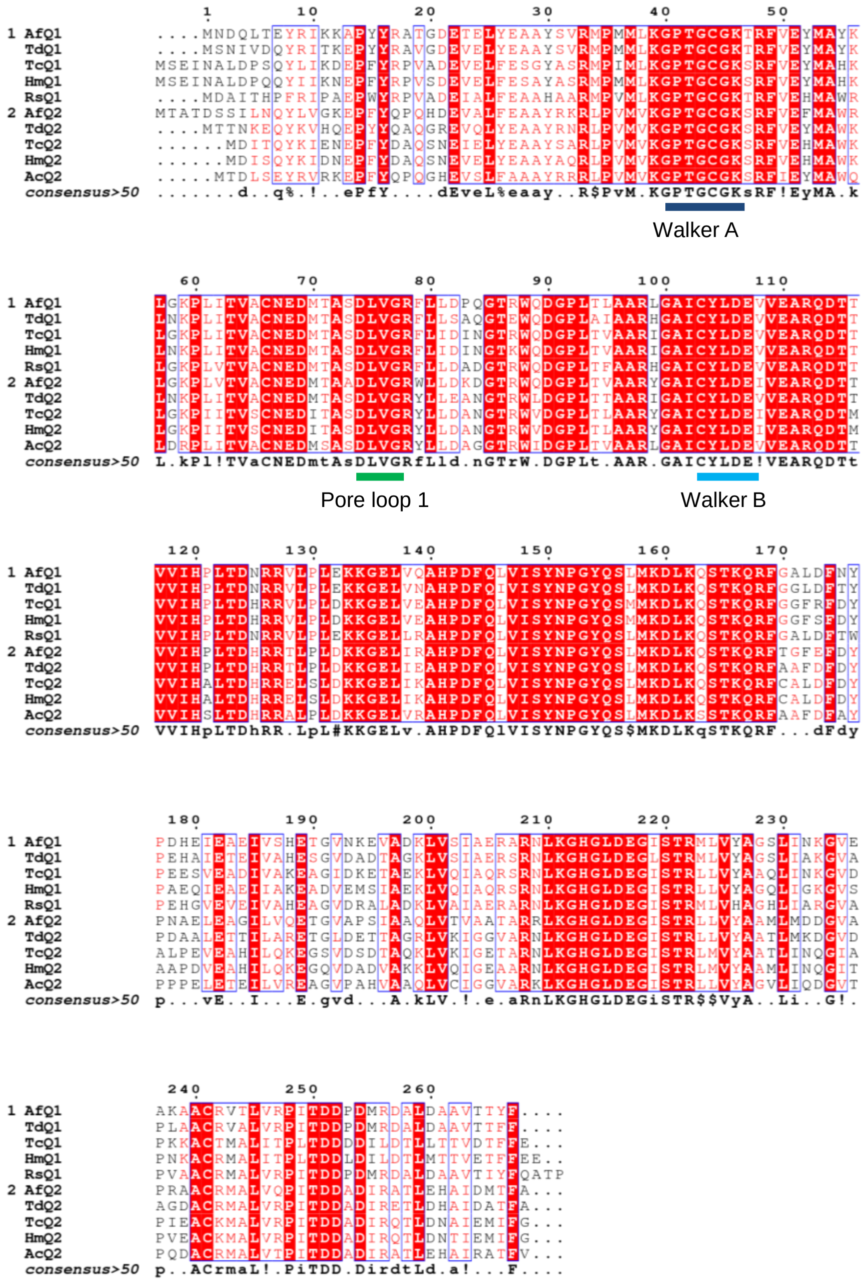

Supplementary Figure 1| Sequence alignment of representative CbbQ sequences. Amino acid sequences of CbbQ1 and CbbQ2 proteins from selected chemoautotrophic bacteria were aligned using Clustal Omega. Red lettering indicates similar residues, whereas identical residues are shown in white on red background. Regions of homology are framed in blue. AAA+ family motifs probed experimentally in this study are indicated below the sequences by coloured bars. Uniprot accession codes of the sequences are: *Acidithiobacillus ferrooxidans*, AfQ1 (B7JA26), AfQ2 (B7J5E4); *Thiobacillus denitrificans*, TdQ1 (Q3SFN1), TdQ2 (Q3SFL6); *Thiomicrospira crunogena* XCL-2, TcQ1 (Q31IJ8), TcQ2 (Q31IK4); *Hydrogenovibrio marinus*, HmQ1 (Q75W43), HmQ2 (Q9WXI1); *Rhodobacter sphaeroides* ATCC17025, RsQ1 (A4WZV3); *Acidithiobacillus caldus*, AcQ2 (F9ZR06). The figure was prepared using Espript[1].

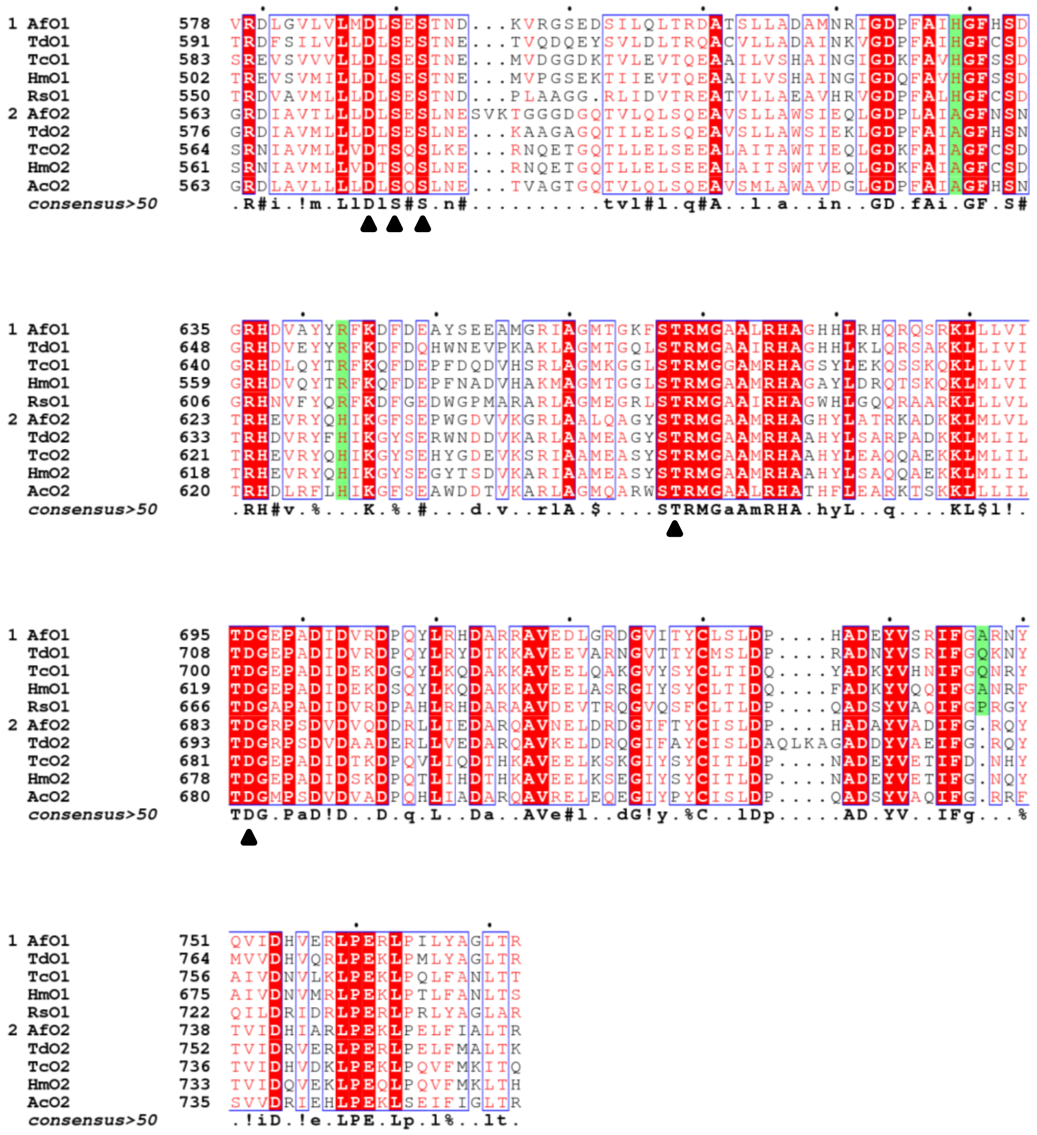

Supplementary Figure 2| Sequence alignment of representative CbbO VWA domain sequences. Amino acid sequences of CbbO1 and CbbO2 proteins from selected chemoautotrophic bacteria were aligned using Clustal Omega, and the C-terminal VWA domain is shown. Red lettering indicates similar residues, whereas identical residues are shown in white on red background. Regions of homology are framed in blue. A green background indicates a systematic difference between O1 and O2 sequences. MIDAS motif residues are indicated by triangles. Uniprot accession codes of the sequences are: *Acidithiobacillus ferrooxidans*, AfO1 (B7JA27), AfO2 (B7J5E5); *Thiobacillus denitrificans*, TdO1 (Q3SFN2), TdO2 (Q3SFL7); *Thiomicrospira crunogena* XCL-2, TcO1 (Q31IJ7), TcO2 (Q31IK5); *Hydrogenovibrio marinus*, HmO1 (Q75W42), HmO2 (Q75W24); *Rhodobacter sphaeroides* ATCC17025, RsO1 (A4WZV4); *Acidithiobacillus caldus*, AcO2 (F9ZR07).

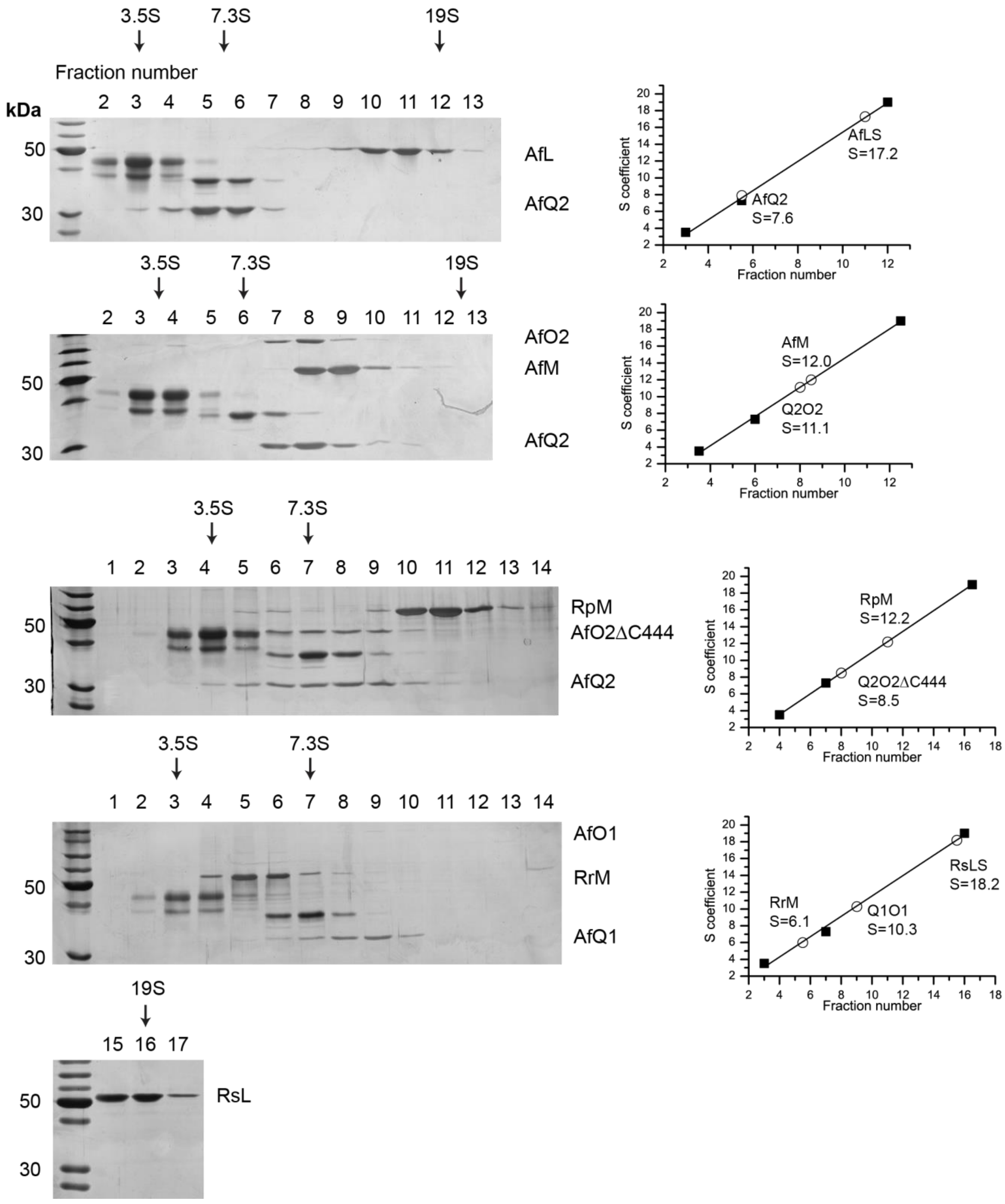

Supplementary Figure 3| Glycerol gradient sedimentation analysis of rubisco and activase proteins. Purified protein complexes were separated using a 10 ml 5-30% glycerol gradient and 500 µl fractions were analyzed using SDS-PAGE followed by silver-staining. Standard proteins used (black squares) were aldolase (7.3S), ovalbumin (3.5S) and thyroglobulin (19S).

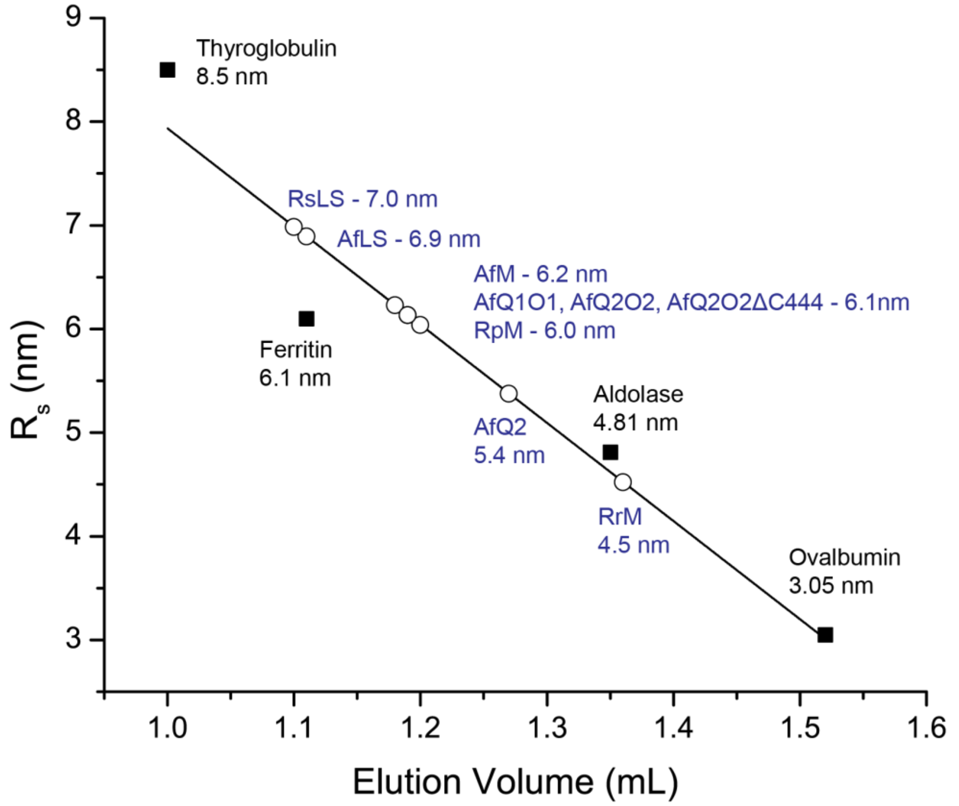

Supplementary Figure 4| Determination of the Stokes radius (R_s). Elution volumes were obtained from the data presented in Figs. 1c and 2a.

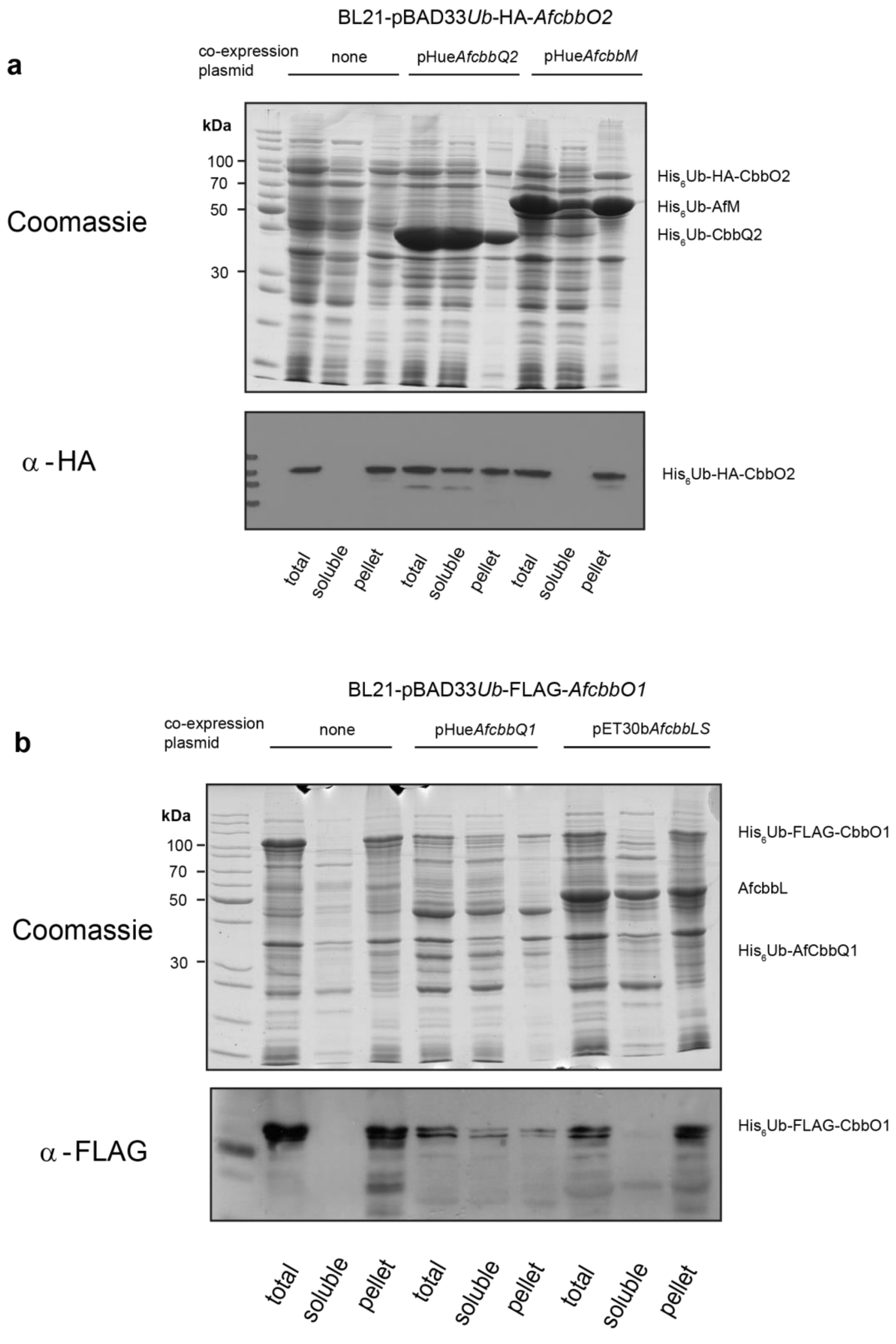

Supplementary Figure 5| Co-expression of CbbQ leads to CbbO solubility in *E. coli*. SDS-PAGE analysis of total, soluble and pellet fractions of cell lysates expressing HA-tagged CbbO2 (a) and FLAG-tagged CbbO1 (b) proteins as His_6-Ubiquitin fusion proteins in the presence or absence of plasmids encoding relevant CbbQ and Rubisco proteins. Gels were analyzed by Coomassie staining (upper panels) and immunoblotting against the HA or FLAG epitope (lower panels).

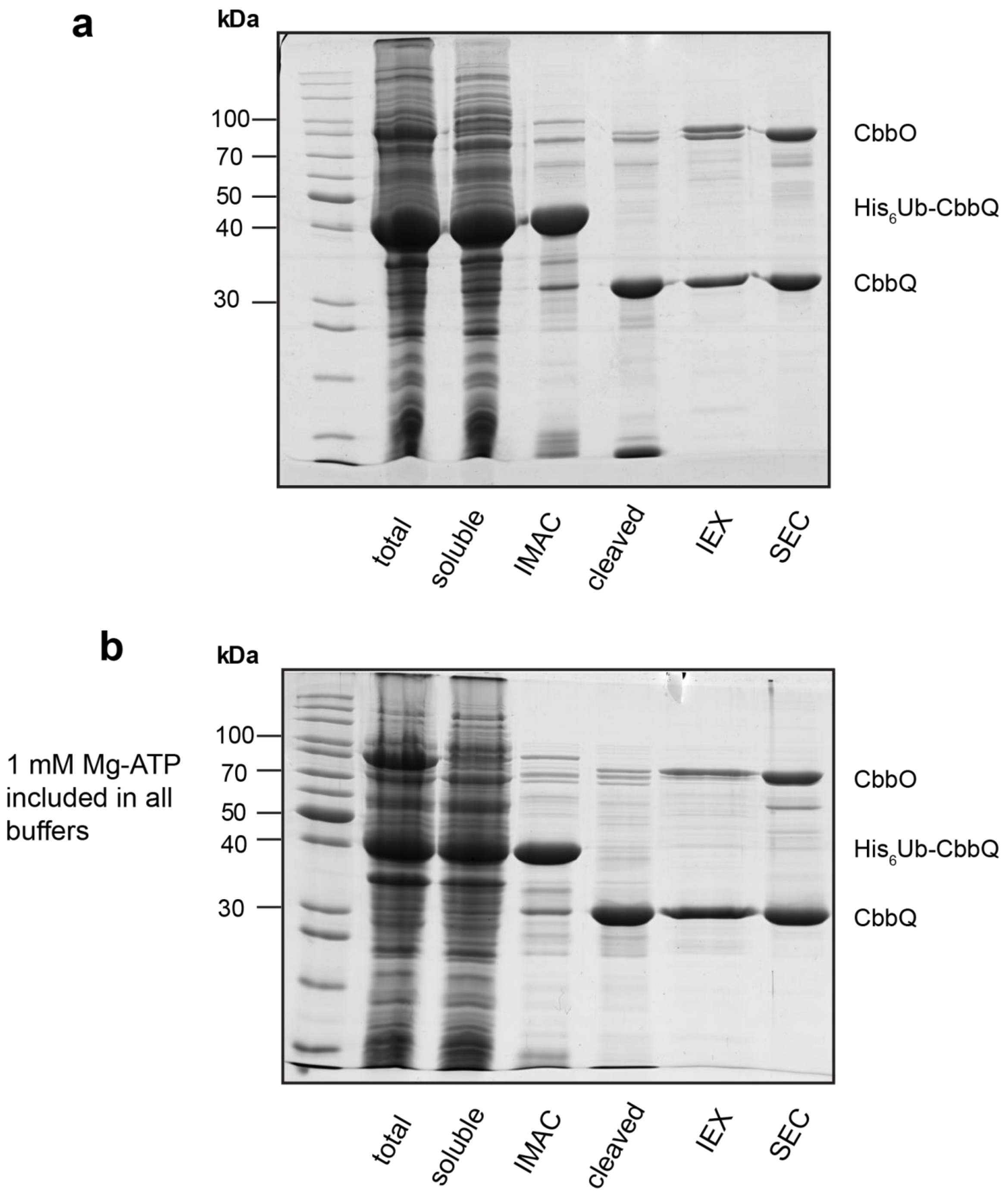

Supplementary Figure 6| Purification of Q2O2. SDS-PAGE analysis of the stages of a representative Q2O2 purification in the absence (a) and presence (b) of 1 mM Mg-ATP in all buffers. IMAC, eluted fraction after immobilized metal affinity chromatography; cleaved, IMAC fraction after cleavage of the His_6-Ub moiety; IEX, ion exchange chromatography; SEC, size exclusion chromatography.

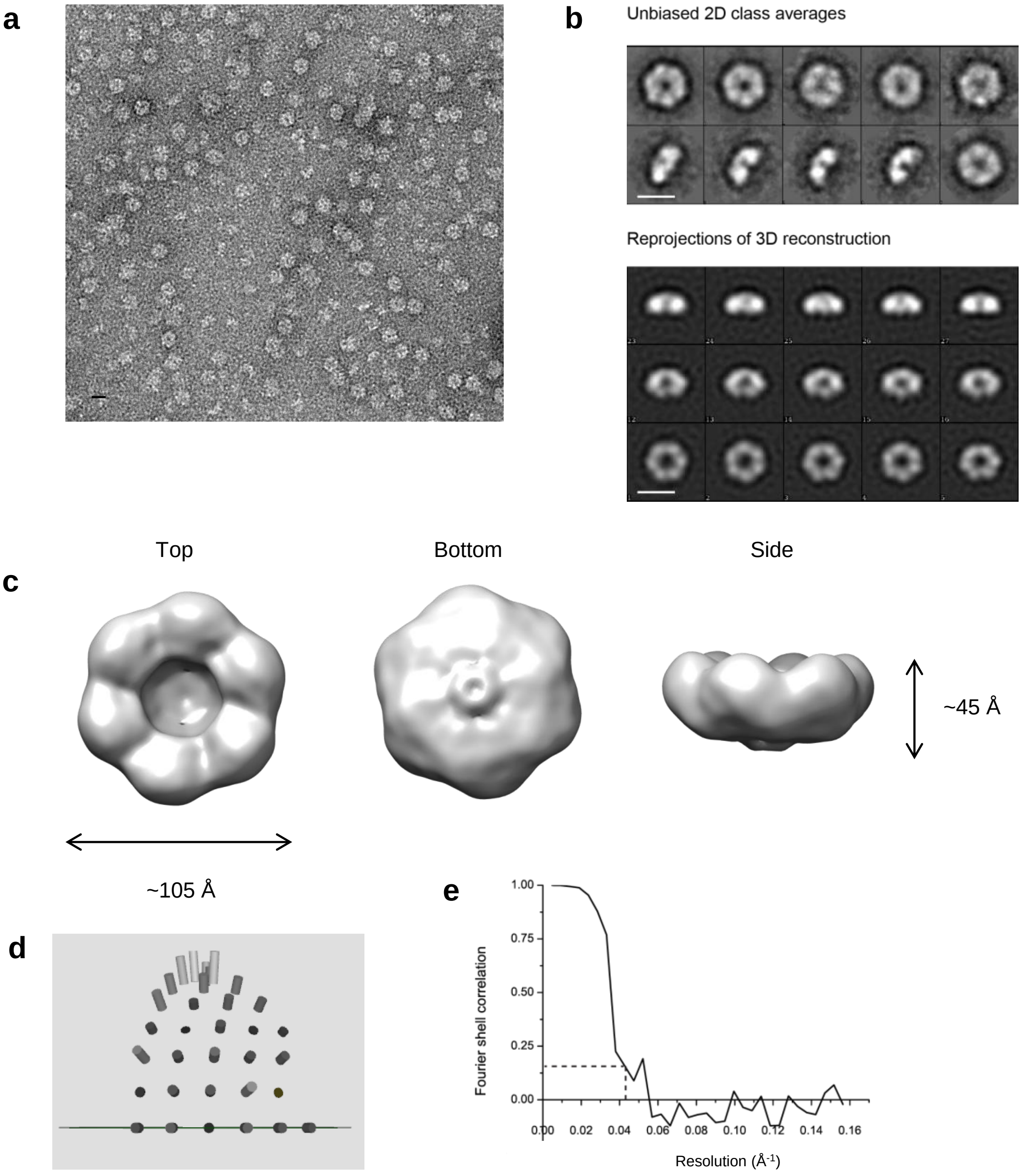

Supplementary Figure 7| Negative-stain electron microscopy of AfQ2. a, A raw electron micrograph of AfQ2 (50 µg/ml) in the presence of 5 mM Mg-ATP. Scale bar, 100 Å. **b,** Comparison of unbiased 2D class averages with re-projections of the 3D-EM reconstruction. **c,** 3D-EM reconstruction of AfQ2 with an imposed six-fold symmetry. The contour level shown is 2.93 corresponding to a volume of 292,000 Å^3. Images were generated using Chimera[2]. **d,** Euler angle distribution of the dataset with six-fold symmetry imposed. The height of the cylinders represents the number of particles present in each orientation (image generated with EMAN2[3]). **e,** Fourier shell correlation curve. The final resolution was estimated at 23 Å resolution using a 0.143 cut-off criterion.

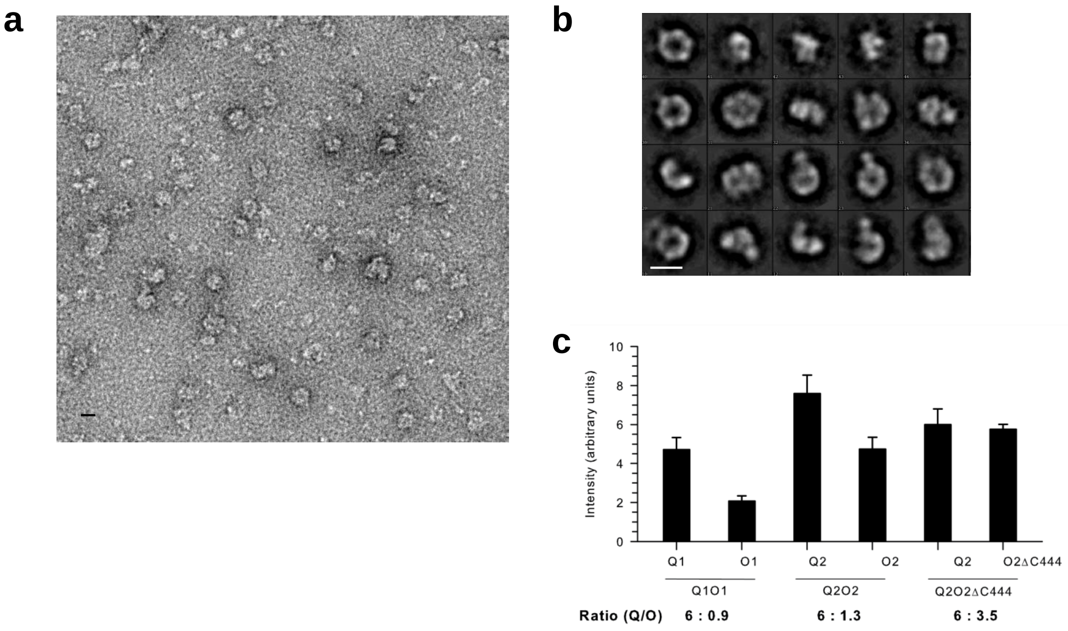

Supplementary Figure 8| EM analysis of Q2O2ΔC444 and quantitative densitometry of the CbbQO complexes. a, A raw electron micrograph of Q2O2ΔC444 (80 µg/ml) in the presence of 5 mM Mg-ATP. **b,** Representative unbiased 2D class averages derived from 12486 particles. Scale bars, 100 Å. **c,** Densitometry of Q1O1, Q2O2 and Q2O2ΔC444. 0.6, 0.8 and 1.0 µg of protein were analyzed by SDS-PAGE and Coomassie-Blue stained bands were quantified using LabQuant. Calculated ratios of CbbQ to CbbO subunits are indicated.

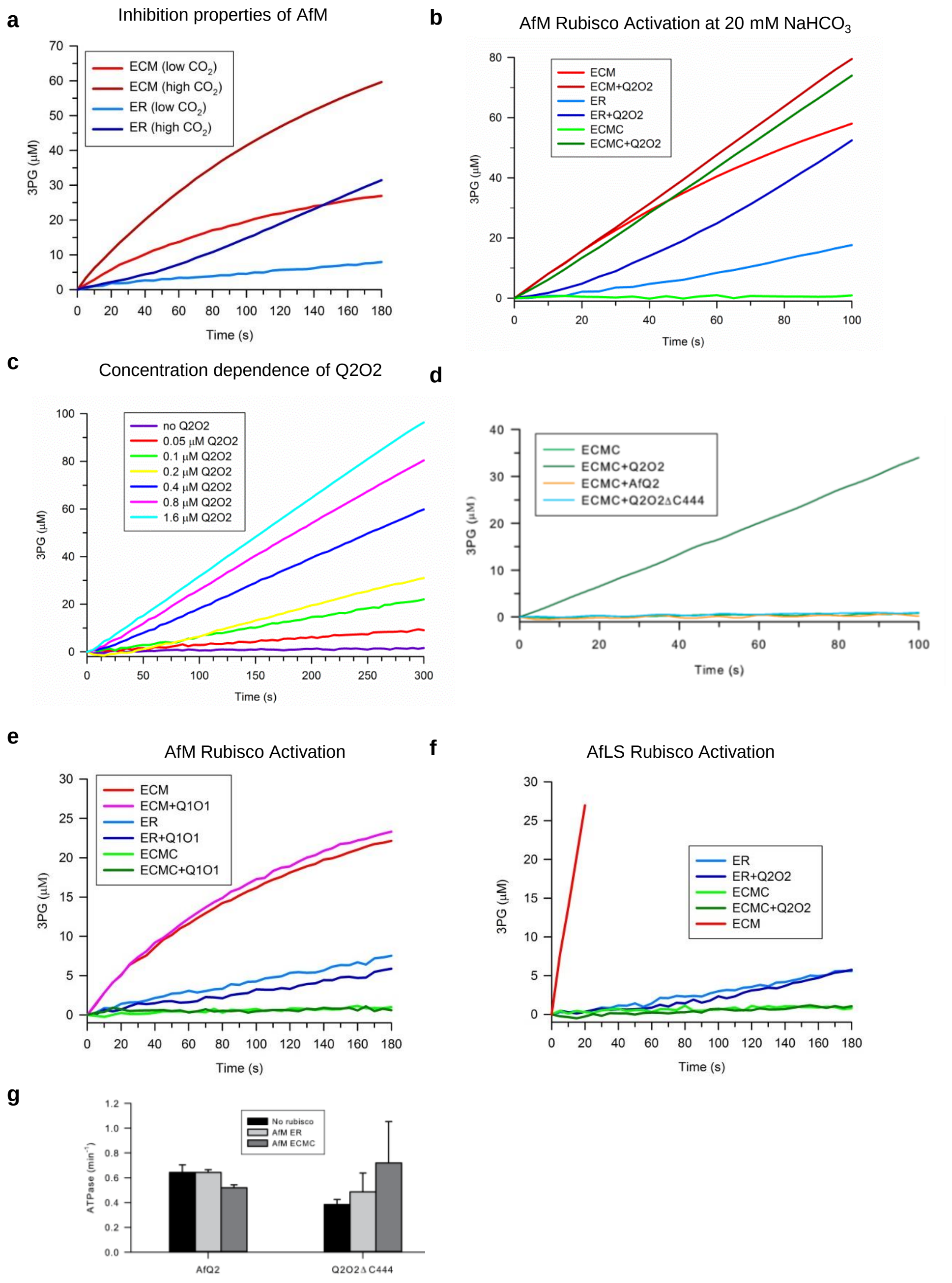

Supplementary Figure 9| Properties of the CbbQO activation systems. a, Inhibition properties of form II rubisco (AfM). Rubisco activity measurements of AfM complexes (0.1 µM active sites) were performed at 5 mM $NaHCO_3$ (low CO_2) and 20 mM $NaHCO_3$ (high CO_2). **b,** Q2O2 also functions at high CO_2 (20 mM $NaHCO_3$) **c,** Activation assays of AfM ECMC were performed at 5 mM $NaHCO_3$ using varying concentrations of Q2O2. **d,** AfQ2 and Q2O2ΔC444 cannot activate AfM. **e,** Form II rubisco (AfM) cannot be activated by Q1O1. **f,** Form I rubisco (AfLS) cannot be activated by Q2O2. Experimental conditions for (b)-(f) are as described for Fig. 3 b-e unless indicated otherwise. **g,** The ATPase activity of AfQ2 and Q2O2ΔC444 is not stimulated by inhibited rubisco. CbbQ protomer concentrations used were 6.6 µM (AfQ2) and 5.2 µM (Q2O2ΔC444). 3 µM of AfM active sites were used. Error bars indicate the mean and S.D. of at least three independent experiments.

a

```
CbbX     73-GNPGTGKT- 28 - LVGQYIGHT - 16 - FIDEAYYL
ClpX    118-GPTGSGKT- 22 - TEAGYVGED - 24 - YIDEIDKI
AfQ1     39-GPTGCGKT- 23 - TASDLVGRF - 24 - YLDEVVEA
AfQ2     43-GPTGCGKS- 23 - TAADLVGRW - 24 - YLDEIVEA
```

b

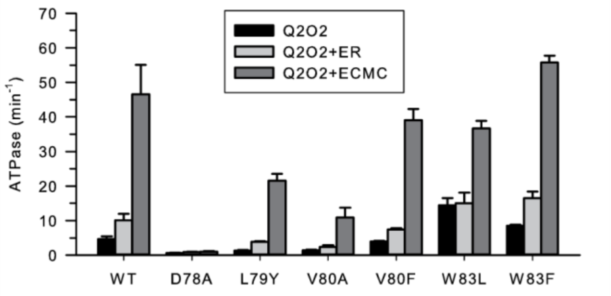

c

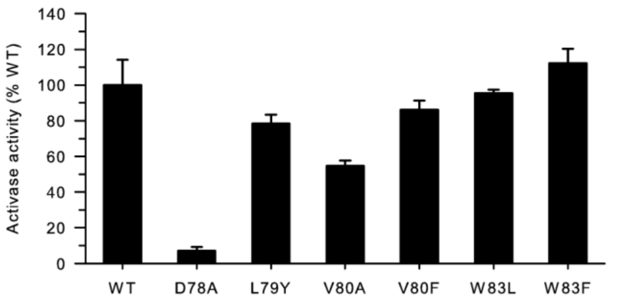

Supplementary Figure 10| The role of pore loop 1 in Q2O2 function. a, Partial sequence alignments of *Rhodobacter sphaeroides* CbbX, *Escherichia coli* ClpX, AfQ1 and AfQ2 to predict pore loop 1, which is located between the Walker A and Walker B motifs. Residues mutated in this analysis are underlined. **b,** ATPase activity of wild-type and mutant Q2O2 proteins (0.27 µM oligomer) in the absence and presence of AfM complexes (3 µM active sites). **c,** Relative rubisco activase activity of wild-type and mutant Q2O2 complexes. Experimental conditions for (b) and (c) are the same as described for Fig. 5. Error bars indicate the mean and S.D. of at least three independent experiments.

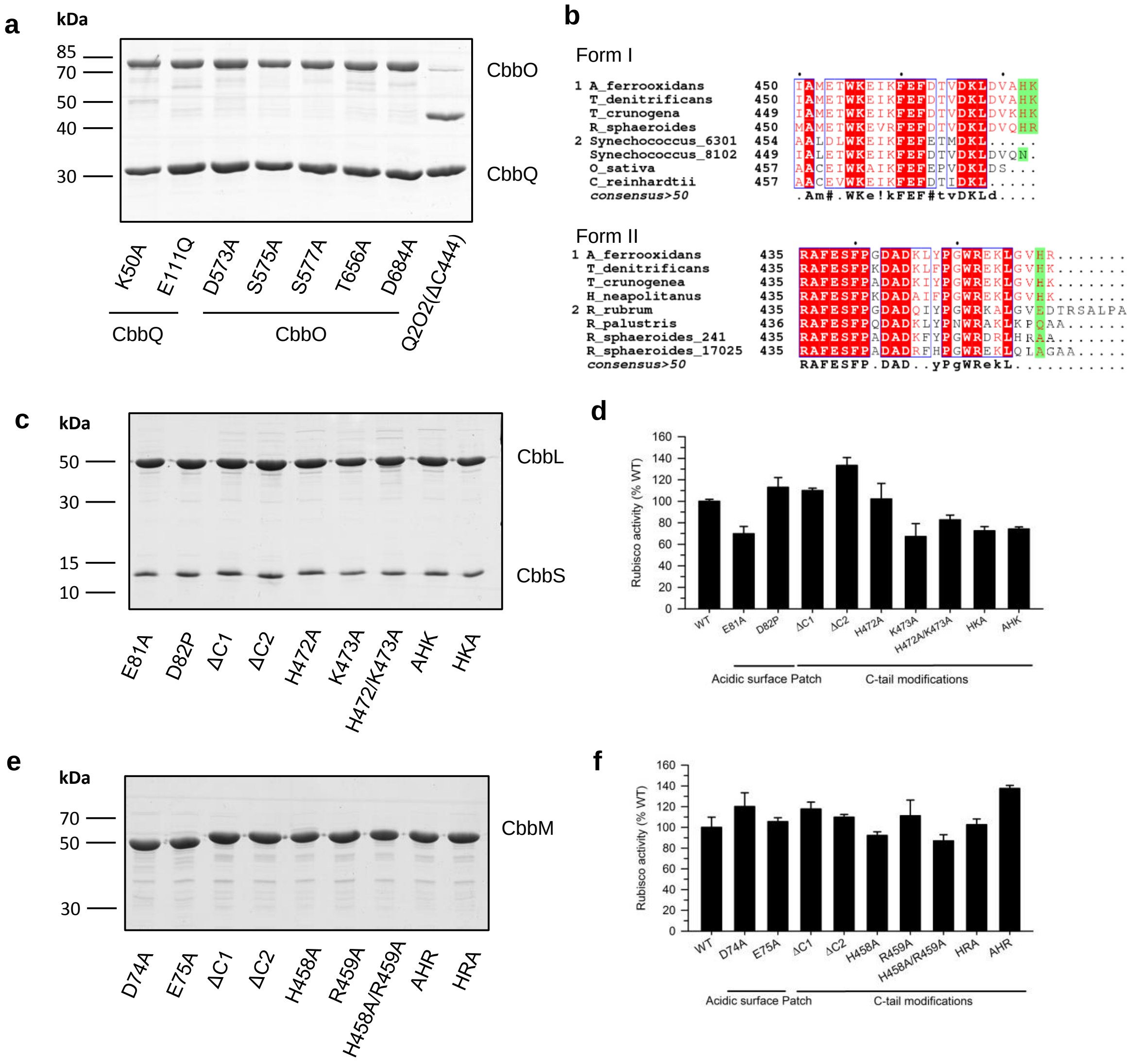

Supplementary Figure 11| Properties of purified Q2O2, AfLS and AfM mutant proteins. **a,c,e** SDS-PAGE analysis of Q2O2 (a), AfLS (c) and AfM (e) mutants. 5 µg (a) and 3 µg (c, e) were loaded per lane. **b,** Sequence alignment of the C-termini of selected form I and form II rubisco large subunits. Group 1 sequences are encoded in *cbbQ-cbbO* containing operons. **d, f** Relative rubisco activities of the fully activated ECM forms of AfLS (d) and AfM (f) mutants. Error bars indicate the mean and S.D. of at least three independent experiments.

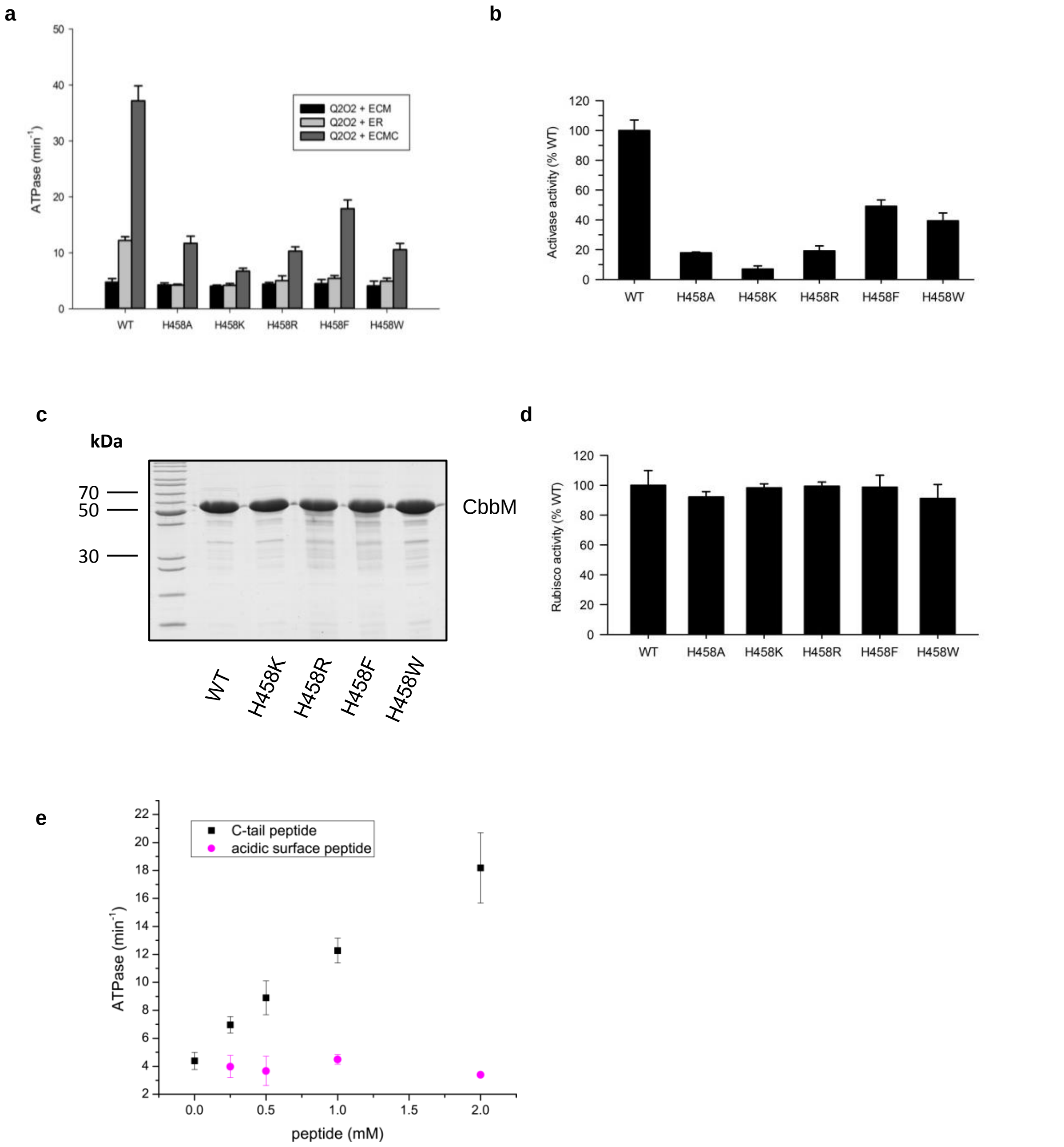

Supplementary Figure 12| The role of the large subunit C-terminus. a, ATPase activity assays of wild-type Q2O2 (0.27 µM oligomer) in the presence of the indicated AfM complexes (3 µM active sites). **b,** Relative rubisco activase activity of Q2O2 using mutant AfM ECMC complexes as substrate. Experimental conditions for (a) and (b) are as in Fig. 5. **c,** SDS-PAGE analysis of the AfM mutants (3 µg protein/lane). **d,** Relative rubisco activities of the fully activated ECM forms of AfM mutants. **e,** A peptide corresponding to the large subunit C-terminus stimulates Q2O2 ATPase activity. The ATPase activity of Q2O2 (0.27 µM oligomer) was measured in the presence of varying concentrations of a C-tail peptide (PGWREKLGVHR) and a peptide including the identified interacting acidic surface residue of AfM (VYHIDEATEDM). Error bars indicate the mean and S.D. of at least three independent experiments.

Supplementary Table 1| Plasmids used in this study.

Name	Relevant information	Source or reference
pHue*AfcbbQ1*	amp^r, T7 promoter	This study
pHue*AfcbbQ2*	amp^r, T7 promoter	This study
pET30b*AfcbbLS*	km^r, T7 promoter	This study
pHue*AfcbbM*	amp^r, T7 promoter	This study
pBAD33*UbAfcbbO1*	cm^r, P_{BAD} promoter	This study
pBAD33*UbAfcbbO2*	cm^r, P_{BAD} promoter	This study
pBAD33*Ub*-FLAG-*AfcbbO1*	cm^r, P_{BAD} promoter	This study
pBAD33*Ub*-HA-*AfcbbO2*	cm^r, P_{BAD} promoter	This study
pTrcrbcM	amp^r, trc promoter	[4]
pHue*RrcbbM*	amp^r, T7 promoter	This study
pHue*RpcbbM*	amp^r, T7 promoter	This study
pET30b*RscbbLS*	km^r, T7 promoter	[5]

Only plasmids containing the wild-type genes are listed for simplicity.

Supplementary Table 2| Primers used in this study.

Primers used to generate *E. coli* expression plasmids.

**Name**	**Sequence (5'→3')**
5SacIIAFCbbQ1	CTCCGCGGTGGTATGAACGACCAGTTGACTGAA
3HindIIIAfCbbQ1	TTAAGCTTTAGAAGTACGTGGTGACTGCGGC
5SacIIAfCbbO1	CTCCGCGGTGGTATGAAACATCTCGAAGAT
3HindIIIAfCbbO1	GTAAGCTTTATCGGGTCAGCCCAGCGTATAAT
5NdeIAfL1	AACATATGGCCGTCAAGACCTATAACGC
3HindIIIAfS1	ATAAGCTTTATACGGTCTTGCCCCGATAGACT
5SacIIAfCbbQ2	CTCCGCGGTGGTATGACTGCAACAGATTCCT
3HindIIIAfCbbQ2	ATAAGCTTTAGGCGAAGGTCATGTCGA
5SacIIAfM	CTCCGCGGTGGTATGGATCAATCTAATCGCTATG
3HindIIIAfM	ATAAGCTTTACCGGTGTACGCCCAGCTTTTCGCGCCAGCCCGGGTAG
5SacIIAfCbbO2	CTCCGCGGTGGTATGACGGATCATCCGCGC
3HindIIIAfCbbO2	ATAAGCTTTACCGGGTCAGGGCGATGAA
pHueFlag_for	CTGGTGTTGCGCCTCCGGGGTGGAATGGATTACAAAGACGATGACGA TAAAGCGGGCCGCGGTGGATCCGAATTCGA
pHueFlag_rev	TCGAATTCGGATCCACCGCGGCCCGCTTTATCGTCATCGTCTTTGTAA TCCATTCCACCCCGGAGGCGCAACACCAG
pHueHA_for	CCTCCGGGGTGGAATGTATCCATATGATGTTCCAGATTATGCTGCGG GCCGCGGTGGAT
pHueHA_rev	ATCCACCGCGGCCCGCAGCATAATCTGGAACATCATATGGATACATT CCACCCCGGAGG
5BamHIRrRbcM	CTGGATCCATGGACCAGTCATCTCGTTACGTCAATCTG
3HindIIIRrRbcM	TGAAGCTTTACGCCGGAAGGGCGCTGC

Primers used to generate mutations in *cbbQ2*

AfQ2K50A_for	AGCGGTTGCGGCGCGTCCCGCTTTG
AfQ2K50A_rev	CAAAGCGGGACGCGCCGCAACCGCT
AfQ2E111Q_for	AGCTATCTGGACCAAATTGTCGAGG
AfQ2E111Q_rev	CCTCGACAATTTGGTCCAGATAGCT
AfQ2D78A_for	ATGACCGCCGCAGCTCTAGTCGGCCGC
AfQ2D78A_rev	GCGGCCGACTAGAGCTGCGGCGGTCAT
AfQ2L79Y_for	ACCGCCGCCGATTATGTCGGCCGCTGG
AfQ2L79Y_rev	CCAGCGGCCGACATAATCGGCGGCGGT
AfQ2V80A_for	CGCCGATCTGGCAGGCCGCTGGCT
AfQ2V80A_rev	AGCCAGCGGCCTGCCAGATCGGCG
AfQ2V80F_for	GCCGCCGATCTGTTTGGCCGCTGGCTG
AfQ2V80F_rev	CAGCCAGCGGCCAAACAGATCGGCGGC
AfQ2W83F_for	CTGGTCGGCCGCTTTCTGCTCGACAA
AfQ2W83F_rev	TTGTCGAGCAGAAAGCGGCCGACCAG
AfQ2W83L_for	CTGGTCGGCCGCTTGCTGCTCGACAA
AfQ2W83L_rev	TTGTCGAGCAGCAAGCGGCCGACCAG

Primers used to generate mutations in *cbbO2*

AfO2D573A_for	CTCCCTGCTGCTGGCCTTGTCCGAATC
AfO2D573A_rev	GATTCGGACAAGGCCAGCAGCAGGGAG
AfO2S575A_for	CTTGCTGGACTTGGCCGAATCACTG
AfO2S575A_rev	CAGTGATTCGGCCAAGTCCAGCAAG

AfO2S577A_for	CTACTTGTCCGAAGCACTGAACGAA
AfO2S577A_rev	TTCGTTCAGTGCTTCGGACAAGTAG
AfO2T656A_for	AAGCCGGTTACTCGGCCCGGATGGGC
AfO2T656A_rev	GCCCATCCGGGCCGAGTAACCGGCTT
AfO2D684A_for	AATGGTACTCACCGCCGGCCGGCCC
AfO2D684A_rev	GGGCCGGCCGGCGGTGAGTACCATT
AfO2(ΔC444)_for	AGGAAGAGGCCTAAGACGCAGAACAG
AfO2(ΔC444)_rev	CTGTTCTGCGTCTTAGGCCTCTTCCT

Primers used to generate mutations in *AfcbbL*

AfLE81A_for	GCATACAGGATTGCAGACGTTCCGGGCGAT
AfLE81A_rev	ATCGCCCGGAACGTCTGCAATCCTGTATGC
AfLD82P_for	CAGGATTGAACCCGTTCCGGGCG
AfLD82P_rev	CGCCCGGAACGGGTTCAATCCTG
AfLΔC1_for	GACGTTGCCCATTAATAAGCACGT
AfLΔC1_rev	ACGTGCTTATTAATGGGCAACGTC
AfLΔC2_for	TGGACGTTGCCTAAAAATAAGCACG
AfLΔC2_rev	CGTGCTTATTTTTAGGCAACGTCCA
AfLH472A_for	CTGGACGTTGCCGCTAAATAAGCACGT
AfLH472A_rev	ACGTGCTTATTTAGCGGCAACGTCCAG
AfLK473A_for	ACGTTGCCCATGCATAAGCACGTTT
AfLK473A_rev	AAACGTGCTTATGCATGGGCAACGT
AfLH472A/K473A_for	CTGGACGTTGCCGCTGCATAAGCACGTTTA
AfLH472A/K473A_rev	TAAACGTGCTTATGCAGCGGCAACGTCCAG
AfLHKA_for	GTTGCCCATAAAGCATAAGCACGTTTAT
AfLHKA_rev	ATAAACGTGCTTATGCTTTATGGGCAAC
AfLAHK_for	CTGGACGTTGCCGCTCATAAATAAGCA
AfLAHK_rev	TGCTTATTTATGAGCGGCAACGTCCAG

Primers used to generate mutations in *AfcbbM*

AfMD74A_for	TCTATCATATCGCCGAAGCCACCGA
AfMD74A_rev	TCGGTGGCTTCGGCGATATGATAGA
AfME75A_for	ATCATATCGACGCAGCCACCGAGGA
AfME75A_rev	TCCTCGGTGGCTGCGTCGATATGAT
AfMΔC1_for	TGGGCGTACACTAATAAAGCTTAGA
AfMΔC1_rev	TCTAAGCTTTATTAGTGTACGCCCA
AfMΔC2_for	AGCTGGGCGTATAACGGTAAAGCTTA
AfMΔC2_rev	TAAGCTTTACCGTTATACGCCCAGCT
3HindIIIAfMH458A	TTAAGCTTCACCGGGCTACGCCCAGCTTT
AfLR459A_for	TGGGCGTACACGCGTAAAGCTTAGAT
AfLR459A_rev	ATCTAAGCTTTACGCGTGTACGCCCA
AfLH458A/R459A_for	AGCTGGGCGTAGCCGCGTAAAGCTTAG
AfLH458A/R459A_rev	CTAAGCTTTACGCGGCTACGCCCAGCT
AfMHRA_for	GGCGTACACCGGGCATAAAGCTTAGAT
AfMHRA_rev	ATCTAAGCTTTATGCCCGGTGTACGCC
3HindIIIAfMAHR	TTAAGCTTTACCGGTGTGCTACGCCCAGCT

AfMH458K_for	AAGCTGGGCGTAAAACGGTAAAGCTTA
AfMH458K_rev	TAAGCTTTACCGTTTTACGCCCAGCTT
AfMH458R_for	AAGCTGGGCGTACGCCGGTAAAGCTTA
AfMH458R_rev	TAAGCTTTACCGGCGTACGCCCAGCTT
AfMH458F_for	AAGCTGGGCGTATTTCGGTAAAGCTTA
AfMH458F_rev	TAAGCTTTACCGAAATACGCCCAGCTT
AfMH458W_for	AAGCTGGGCGTATGGCGGTAAAGCTTA
AfMH458W_rev	TAAGCTTTACCGCCATACGCCCAGCTT

Supplementary References

1 Gouet, P., Courcelle, E., Stuart, D. I. & Metoz, F. ESPript: analysis of multiple sequence alignments in PostScript. *Bioinformatics* **15**, 305-308 (1999).

2 Pettersen, E. F. *et al.* UCSF Chimera--a visualization system for exploratory research and analysis. *J Comput Chem* **25**, 1605-1612 (2004).

3 Tang, G. *et al.* EMAN2: an extensible image processing suite for electron microscopy. *J. Struct. Biol.* **157**, 38-46 (2007).

4 Mueller-Cajar, O., Morell, M. & Whitney, S. M. Directed Evolution of Rubisco in Escherichia coli Reveals a Specificity-Determining Hydrogen Bond in the Form II Enzyme. *Biochemistry* **46**, 14067-14074 (2007).

5 Mueller-Cajar, O. *et al.* Structure and function of the AAA+ protein CbbX, a red-type Rubisco activase. *Nature* **479**, 194-199 (2011).
